# circular RNA circ-231 promotes protein biogenesis of TPI1 and PRDX6 through mediating the interaction of eIF4A3 with STAU1 to facilitate unwinding of secondary structure in 5′ UTR, enhancing progression of human esophageal squamous cell carcinoma (ESCC)

**DOI:** 10.7150/jca.92578

**Published:** 2024-03-11

**Authors:** Guo-Wei Huang, Ti-Qun Yang, Qian-Qian Chen, Xing-Mu Liu, Ling-Hui Xie, Wei Huang, Xue-Ling Chen, Yi-Qun Geng, Jiang Gu

**Affiliations:** 1 Department of Pathology, Shantou University Medical College, Shantou, 515041, Guangdong, P.R. China.; 2 Provincial Key Laboratory of Infectious Diseases and Molecular Immunopathology, Shantou University Medical College, Shantou, 515041, Guangdong, P.R. China.; 3 Department of Surgery, Second Affiliated Hospital, Shantou University Medical College, Shantou, 515041, Guangdong, P.R. China.

**Keywords:** circ-231, RNA binding protein, eIF4A3, STAU1, Interaction

## Abstract

**Background:** The nuclear cap-binding complex (CBC)-dependent translation (CT) is an important initial translation pathway for 5′-cap-dependent translation in normal mammal cells. Eukaryotic translation initiation factor 4A-III (eIF4A3), as an RNA helicase, is recruited to CT complex and enhances CT efficiency through participating in unwinding of secondary structure in the 5′ UTR. However, the detailed mechanism for eIF4A3 implicated in unwinding of secondary structure in the 5′ UTR in normal mammal cells is still unclear. Specially, we need to investigate whether the kind of mechanism in normal mammal cells extrapolates to cancer cells, e.g. ESCC, and further interrogate whether and how the mechanism triggers malignant phenotype of ESCC, which are important for identifying a potential therapeutic target for patients with ESCC.

**Methods:** Bioinformatics analysis, RNA immunoprecipitation and RNA pulldown assays were performed to detect the interaction of circular RNA circ-231 with eIF4A3. *In vitro* and *in vivo* assays were performed to detect biological roles of circ-231 in ESCC. RNA immunoprecipitation, RNA pulldown, mass spectrometry analysis and co-immunoprecipitation assays were used to measure the interaction of circ-231, eIF4A3 and STAU1 in HEK293T and ESCC. *In vitro* EGFP reporter and 5′ UTR of mRNA pulldown assays were performed to probe for the binding of circ-231, eIF4A3 and STAU1 to secondary structure of 5′ UTR.

**Results:** RNA immunoprecipitation assays showed that circ-231 interacted with eIF4A3 in HEK293T and ESCC. Further study confirmed that circ-231 orchestrated with eIF4A3 to control protein expression of TPI1 and PRDX6, but not for mRNA transcripts. The in-depth mechanism study uncovered that both circ-231 and eIF4A3 were involved in unwinding of secondary structure in 5′ UTR of TPI1 and PRDX6. More importantly, circ-231 promoted the interaction between eIF4A3 and STAU1. Intriguingly, both circ-231 and eIF4A3 were dependent on STAU1 binding to secondary structure in 5′ UTR. Biological function assays revealed that circ-231 promoted the migration and proliferation of ESCC via TPI1 and PRDX6. In ESCC, the up-regulated expression of circ-231 was observed and patients with ESCC characterized by higher expression of circ-231 have concurrent lymph node metastasis, compared with control.

**Conclusions:** Our data unravels the detailed mechanism by which STAU1 binds to secondary structure in 5′ UTR of mRNAs and recruits eIF4A3 through interacting with circ-231 and thereby eIF4A3 is implicated in unwinding of secondary structure, which is common to HEK293T and ESCC. However, importantly, our data reveals that circ-231 promotes migration and proliferation of ESCC and the up-regulated circ-231 greatly correlates with tumor lymph node metastasis, insinuating that circ-231 could be a therapeutic target and an indicator of risk of lymph node metastasis for patients with ESCC.

## Introduction

The pathway of mRNA initial translation contains 5′-cap-dependent or 5′-cap-independent translation in normal mammal cells. The nuclear cap-binding complex (CBC)-dependent translation (CT) is of decisive importance for 5′-cap-dependent initial translation in normal mammal cells. Eukaryotic translation initiation factor 4A-III (eIF4A3), as a member of DEAD protein family (including eIF4AI, II, III) with putative RNA helicase activity, was previously shown as a part of exon-junction complex (EJC), which took part in pre-mRNA, circular RNA alternative splicing and stabilized mRNAs 1-3. However, some evidence indicated that eIF4A3 was involved in mRNA translation. For example, eIF4A3 promoted tumor growth through controlling protein biogenesis in ribosome 4. eIF4A3 impeded selenoprotein translation through interacting with a selenocysteine (Sec) insertion sequence (SECIS) in 3′ UTR 5. Importantly, eIF4A3 was recruited to CT complex for enhancing CT efficiency through participating in unwinding of secondary structure in the 5′ UTR, further validating eIF4A3 participating in mRNA translation 6. However, the mechanism for how eIF4A3 interacts with secondary structure is still unclear in normal mammal cells. Elucidating the detailed mechanism by which eIF4A3 unwinds secondary structure in cancer cells is especially important to identify a potential therapeutic target. Dudekula DB, *et al*. predicted that RNA binding proteins (RBPs) interacted with circRNA junctions and eIF4A3 possessed a preference for binding to circRNA junctions, compared to other RBPs 7. Their results predicted that 117,000 circRNAs could potentially associate with eIF4A3 and 9,945 of all circRNAs were predicted to be targeted by eIF4A3 in their junction sequence 7, which prompted us to explore whether eIF4A3 interacts with circRNAs to unwind secondary structure in the 5′ UTR in normal and cancer cells.

**Figure 1:**
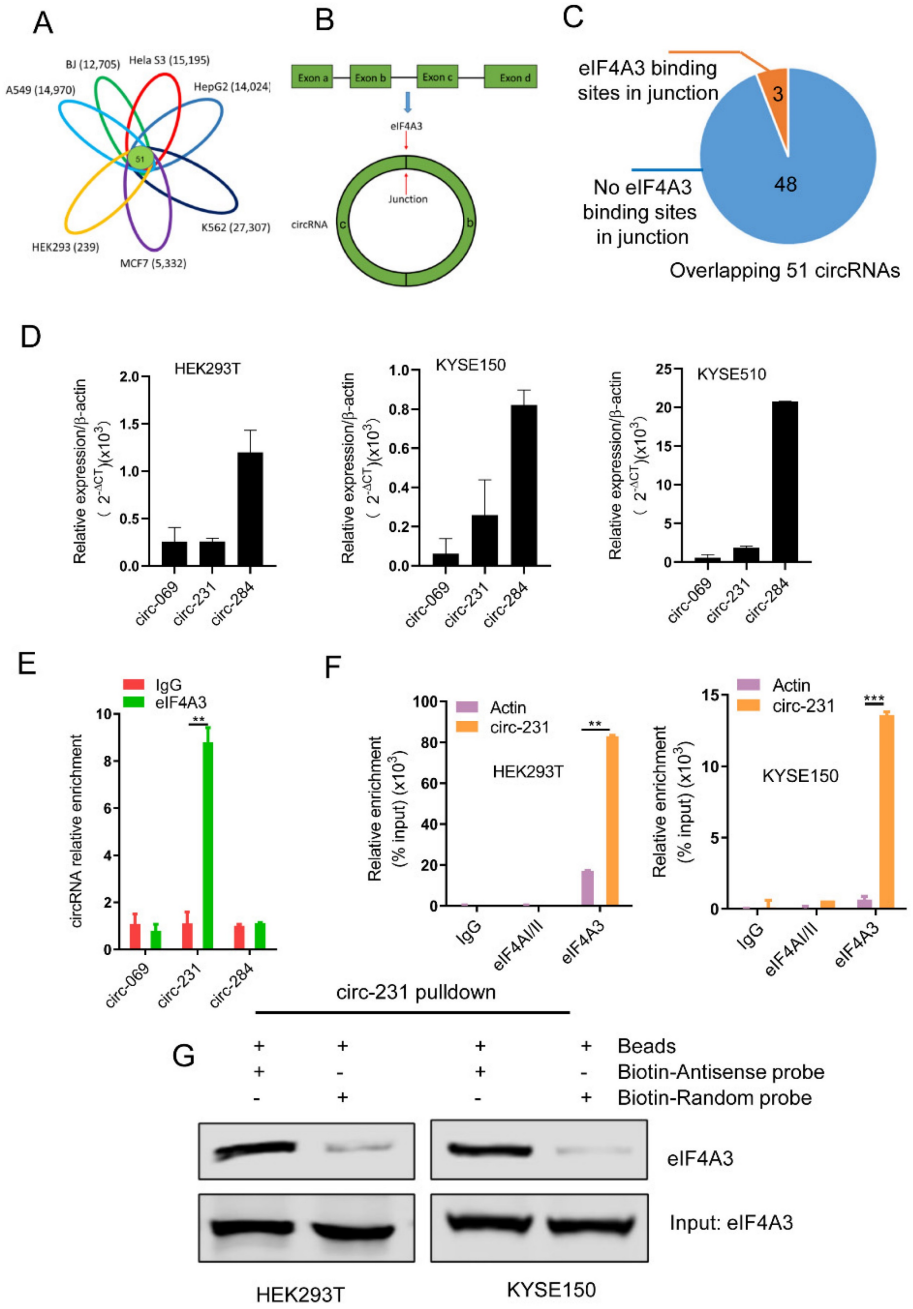
circ-231 interacts with eIF4A3. (**A**) The overlapped circRNAs were counted using R package gplots in seven cell lines, including A549, BJ, Hela S3, HepG2, K562, MCF7 and HEK293 cells. (**B and C**) The number of circRNAs in total 51 overlapped circRNAs containing eIF4A3-binding sites were analyzed and counted according to Circinteractome database. (**D**) circ-231, circ-069 and circ-248 were measured by qRT-PCR. β-actin used as internal control. (**E**) Total protein from HEK293T cells was extracted and 10% extracts were selected as input. Mouse anti-eIF4A3 or normal mouse IgG were incubated with protein extracts overnight at 4°C. RNA from the precipitated complex was extracted and circ-231, circ-069 and circ-248 were determined using qRT-PCR. Values are mean ± standard deviation (SD). ^*^P˂0.05, ^**^P<0.01. (**F**) RNA immunoprecipitation assays for eIF4A3 in HEK293T, KYSE150 cells were performed according to the method described in (**E**). Mouse anti-eIF4AI/II and normal IgG were selected as negative control. The relative enrichment of circ-231 was detected using qRT-PCR. Values are mean ±SD.^ *^P˂0.05, ^**^P<0.01, ^***^P<0.001. (**G**) circ-231 pulldown assays were performed. Biotin-labeled DNA probe complementary to junction sequence of circ-231 or random probe were incubated with HEK293T or KYSE150 cell extracts at 4°C overnight and the biotin-labeled probes were pulldown by streptavidin-coupled magnetic beads and eIF4A3 was determined with western blot.

Esophageal cancer (EC) has gradually become a disease seriously threatening health of human beings across the world and the annual new cases of EC possesses the seventh place, whereas its morbidity is ranked as the sixth place among all the malignant tumors. The study indicated that incident rate of EC in Eastern Asia, Southern and Eastern Africa was distinctly higher than that in other regions in the worldwide 8. Specially, in China, a large number of patients diagnosed as ESCC, died from the malignancy because of lack of effective diagnosis and treatment modality. Publicly available data is indicative of dysregulated circular RNAs dictating malignant phenotype, for example the proliferation and metastasis of tumors, underscoring the feasibility of circRNAs as a vulnerable target for tumor therapy 9. In the present study, we found that eIF4A3 interacted with circ-231 in HEK293T cells and ESCC (**Fig. 1**), enlightening us to explore whether eIF4A3 unwinds secondary structure through interacting with circ-231 and whether the mechanism is common to HEK293T cells and ESCC, which is significant to identify a potential therapeutic target in ESCC. Recently, partial evidence has documented that circ-231 participates in tumor progression. For instance, circ-231 acts as a sponge for miR-375 or binds to IGFBP2 to maintain the expression of CCND2, thereby exacerbating progression of colorectal cancer 10. Another study shows that m^6^A-modified circ-231 interacts with IGFBP2 and binds to FOXM1 mRNA and further maintains the stability of FOXM1 mRNA, facilitating progression of cervical cancer 11. Intriguingly, cicr-231 was most remarkably expressed across acquired circRNAs derived from circRNA profile chips in both colorectal cancer and cervical cancer, indicating that circ-231 might be essential for progression of a multitude of malignant tumors. Moreover, to date, no more report about circ-231 in other malignancy, rather than colorectal cancer and cervical cancer, has been available, which enlightens us to further interrogate the biological function of circ-231 in ESCC, since ESCC has a very high incidence in Chaoshan area in Shantou, China and there has been no effective diagnosis and treatment for patients with ESCC.

In the present study, we found that eIF4A3 collaborated with circ-231 and STAU1 to post-transcriptionally modulate protein synthesis of TPI1 and PRDX6 through unwinding of 5′ UTR in HEK293T cells and ESCC. More importantly, circ-231 promoted migration and proliferation of ESCC via TPI1 and PRDX6 by the aforementioned mechanism and ESCC with highly-expressed circ-231 was prone to lymph node metastasis. Our data delineates a new mechanism for eIF4A3 interacting with circ-231 to implicate in unwinding of 5′ UTR, and meanwhile our data throws light on circ-231 to be a therapeutic target and an indicator for risk of lymph node metastasis for patients with ESCC.

## Materials and Methods

### Tissue samples and cell culture

Paired tumor and adjacent non-tumor tissues were collected from patients with ESCC and all the tissues were snap frozen in liquid nitrogen after being resected and then stored at - 80 °C until RNA being extracted. This study was approved by the Ethical Committee of the Central Hospital of Shantou City and the Medical College of Shantou University (SUMC-2019-08) and written informed consent was obtained from all surgical patients to use resected samples and clinical data for research. In the present study, tumor samples dissected from patients with ESCC were included if patients had no concurrent autoimmune disease and metabolic disease. KYSE150 and KYSE510, human esophageal cancer cell lines, were cultured in RPMI-1640 medium containing 10% fetal bovine serum (FBS). HEK293T cell line was kindly provided by Professor Dong Xie (The Institute for Nutritional Sciences, Chinese Academy of Sciences, China) and HepG2 cell line was stored in our lab and the two cell lines were maintained in Dulbecco's modified Eagle's medium (DMEM) containing 10% FBS.

### Construction of plasmids

For the pTPI1 and pPRDX6 plasmids, the 5′ UTRs of TPI1 and PRDX6 were cloned into the Hind *III* site upstream of the EGFP initiation codon in pcDNA3.1-EGFP. The stem-loop structure, denoted as SS1, was used as a positive control and inserted into the Hind *III* site of pcDNA3.1-EGFP for the pSS1 plasmid. Plasmid construction was performed by Genewiz (Suzhou, China). The sequences were shown in Supplementary method in Supplementary file. TPI1 and PRDX6 protein expression vectors were purchased from Sino Biological, Inc. (Beijing, China). Expression vectors for pcDNA3.1-Flag-eIF4A3 full-length or deletion mutants, pcDNA3.1-HA-STAU1 full-length or deletion mutants and plasmids of pLKO-Tet-On-shRNA-Scramble, pLKO-Tet-On-shRNA1-circ-231, pLKO-Tet-On-shRNA2-circ-231, pLKO-shRNA1-circ-231, pLKO-shRNA2-circ-231 and pLKO-shRNA-Scramble were constructed by IGE Biotechnology Co., Ltd. (Guangzhou, China). The sequences of shRNA-circ-231 or Scramble inserted into pLKO-Tet-On empty vectors were shown in Supplementary method. The method for construction of circ-231 expression vector was described in Supplementary method.

### Real-time RT-PCR and western blot

Real-time RT-PCR and western blot were performed as previously reported 12. Briefly, total RNA was extracted and according to the manufacturer's instructions, cDNA synthesis was performed using a PrimeScript™ RT reagent kit and real-time PCR was performed using a SYBR® Premix Ex Taq™ kit (Takara, Dalian, China). Primer sequences are shown in Supplementary Table S1. As for western blot, mouse anti-eIF4A3, mouse anti-STAU1, mouse anti-TPI1, mouse anti-PRDX6, mouse anti-human β-actin (Santa Cruz Biotechnology, Santa Cruz, CA, USA), rabbit anti-HA or Flag (Proteintech, Wuhan, China) were used as the primary antibodies and IRDye 680 goat anti-mouse or anti-rabbit IgG were the secondary antibody (LI-COR Biosciences, Lincoln, NE, USA). Protein relative levels were measured with the LI-COR Odyssey Infrared Imaging System (LI-COR Biosciences, Lincoln, NE, USA).

### Establishment of shRNA- or Tet-On-mediated shRNA-expressing cells

For establishment of pLKO-shRNA1-circ-231, pLKO-shRNA2-circ-231 and pLKO-shRNA-Scramble expressing cells, plasmids of pLKO-shRNA1-circ-231, pLKO-shRNA2-circ-231 and pLKO-shRNA-Scramble were individually co-transfected with packaging vectors into HEK293T cells and viral particles were collected and filtered with 0.45 μm filter after 48 h and then viral particles were added to KYSE150 and KYSE510 cells. The next day, 500 ng/ml puromycin was added to the cells to screen shRNA-expressing cells. According to the above method, both KYSE150 and KYSE510 cells expressing pLKO-Tet-On-shRNA-Scramble, pLKO-Tet-On-shRNA1-circ-231 and pLKO-Tet-On-shRNA2-circ-231 were also individually established. All the cells were subjected to five or six passage and circ-231 relative levels were measured with real-time RT-PCR. As for Tet-On-mediated shRNA-expressing cells, doxycycline (dox) (Beyotime, Suzhou, China) with 2 μg/ml was *in vitro* used to induce the expression of shRNA1-circ-231, shRNA2-circ-231 and shRNA-Scramble and then the expression of circ-231 was measured with qRT-PCR.

### Cell migration and colony formation assays

Cell migration assays were performed as previously described 13. Briefly, cells were starved for 18 h in medium without serum and the next day, 5x10^4^ cells were plated in medium without serum in the upper chamber (24-well insert; pore size, 8 µm; BD Biosciences) and in the lower chamber, medium supplemented with 10% FBS was added. After 48 h, cells were fixed and stained with haematoxylin solution (Thermo Fisher Scientific, San Jose, CA, USA). Those cells which did not migrate through the pores were removed with a cotton swab and the images of the cells that migrated through the pores were photographed with a Leica DM2000 microscope (Leica Microsystems, Wetzlar, Germany) and the number of cells derived from at least 20 views in each group were counted and analyzed. For colony formation assays, cells were plated in a six-well plate and colonies were stained with haematoxylin solution and counted.

### Isolation of cytoplasmic and nuclear extracts

Cell cytoplasmic and nuclear extracts were prepared according to the described protocol (http://www.lifetechnologies.com/cn/zh/home/references/protocols/cell-and-tissue-analysis/elisa-protocol/elisa-sample-preparation-protocols/nuclear-extraction method-.html). RNA from the cytoplasmic extract was isolated according to the instruction of the kit (DP419, Tiangen Biotech, Beijing, China) and RNA from the nuclear pellet was extracted using TRizol (Thermo Fisher Scientific, San Jose, CA, USA). circ-231 was detected with real time RT-PCR and nuclear lncRNA MALAT1 were served as the nuclear control transcript 14. Primers for PCR are shown in Supplementary Table S1.

### RNA fluorescence *in situ* hybridization (RNA FISH) and immunofluorescence co-localization

T7 RNA polymerase binding site sequence was added to 5′ end of forward primer for circ-231 antisense probe synthesis and 5′ end of reverse primer for sense probe synthesis. Primer sequences were shown in Supplementary Table S1. circ-231 expression vector was used as PCR template and the PCR products were purified and used as *in vitro* transcription templates using a T7 RNA polymerase kit (Takara, Dalian, China). Both transcripts were labeled with biotin-UTP (Roche, Basel, Switzerland) and subsequently used in RNA FISH for HEK293T cells or human esophageal cancer tissues according to the previous reports 12, 13. For co-localization of circ-231 and eIF4A3, after probe hybridization, tissue sections were washed with 2 × SSC containing 50% formamide and 2 × SSC twice at 37 °C for 15 min and then incubated with mouse anti-eIF4A3 for 2 h at 37°C. After washing with PBS, Cy3-conjugated streptavidin (Thermo Fisher Scientific, San Jose, CA, USA) and Alexa Fluor 488 dye-coupled goat anti-mouse IgG (Thermo Fisher Scientific, San Jose, CA, USA) were incubated with tissue sections for 1 h at room temperature. After DAPI staining, fluorescent images were taken with a fluorescence microscope Axio Imager A2 (Zeiss, Bochum, Germany) or laser scanning confocal microscope Fluoview FV1000 (Olympus, Tokyo, Japan).

### RNA Immunoprecipitation (RIP) and co-immunoprecipitation (co-IP)

RNA immunoprecipitation assays were performed as previously described 12. Briefly, cells were lysed in lysis buffer (50 mM Tris-HCl (pH7.5), 150 mM NaCl, 5mM EDTA, 0.5% NP-40 and 1% Triton X-100) and 10% lysates were used as input and the left lysates were individually incubated with mouse anti-eIF4A3, mouse anti-eIF4AI/II, rabbit anti-Flag or HA antibodies and normal mouse IgG at 4°C overnight. The next day, the complexes were immunoprecipitated by protein A/G magnetic beads (Thermo Fisher Scientific, San Jose, CA, USA) and RNA was extracted from immunoprecipitates using Trizol reagent (Thermo Fisher Scientific, San Jose, CA, USA). Subsequently, circ-231 was measured with real-time RT-PCR. IgG was set as the negative control. For co-immunoprecipitation, cells were lysed in buffer (50 mM Tris-HCl (pH7.5), 150 mM NaCl, 0.5%NP-40), mouse anti-STAU1, mouse anti-eIF4A3 and rabbit anti-HA were used as primary antibodies (Santa Cruz Biotechnology, Santa Cruz, CA, USA) and the complex immunoprecipitated with protein A/G magnetic beads was determined with western blot.

### RNA pulldown assays

circ-231 pulldown assays were performed according to the method reported previously 15 with slight modifications. Briefly, DNA probe complementary to the junction region of circ-231 or random control probe was synthesized and labeled with biotin at 5′ end (Sangon Biotech, Shanghai, China). The antisense or random probe sequence was as follows: biotin-5′-AGTATCACATTTAAACCCTTATCTGTTCAGTGGAG-3′ (the probe antisense to circ-231); Biotin-5′-AAACAGTACTGGTGTGTAGTACGAGCTGAAGCTAC-3′ (the random probe). The probes were dissolved in 500 μl binding buffer (0.5 M NaCl, 20 mM Tris-HCl, pH 7.5, 1 mM EDTA pH 8.0) supplemented with 100 μg/ml ssDNA, 100 μg/ml tRNA and 400 μg/ml BSA (Thermo Fisher Scientific, San Jose, CA, USA) and incubated with streptavidin-coupled magnetic beads (Thermo Fisher Scientific, San Jose, CA, USA) at room temperature for 3 h and probe-coated magnetic beads were washed with binding buffer. HEK293T cells were lysed in buffer (50 mM Tris-HCl (pH7.5), 150 mM NaCl, 5mM EDTA, 0.5% NP-40 and 1% Triton X-100) and 1.5 mg total protein was incubated with probe-coated magnetic beads at 4°C overnight and eIF4A3 or STAU1 from the immunoprecipitated protein was determined with western blot. For pulldown assays for secondary structure at 5′ end of TPI1 and PRDX6 mRNAs, DNA oligo fragments from 5′ UTR of the two genes, containing T7 RNA polymerase binding site sequence, were synthesized, and annealed to form a double-strand to be used as the template for the synthesis of biotin-labeled RNA with *in vitro* transcription according to the procedure of biotin labeling RNA mix (Roche, Basel, Switzerland). The synthesized RNA was purified with RNA miniprep kit (Biomiga, San Diego, CA) and 1μg biotinylated RNA was used to form the secondary structure in RNA structure buffer according to the previously reported method 16 and incubated with 1.5 mg protein extract from HEK293T cells with STAU1, eIF4A3 or circ-231 knockdown at 4°C overnight. eIF4A3 or STAU1 from the immunoprecipitated complex was determined with western blot and circ-231 from the complex was measured by using qRT-PCR.

### Coomassie blue staining and liquid chromatography coupled with hybrid quadrupole-time of flight (LCMS-Q-TOF)

siRNAs against circ-231, eIF4A3 or Scramble were individually transfected into HEK293T cells. After 48 h, protein from each of groups was extracted and the concentrations of protein were measured using a BCA kit (Thermo Fisher Scientific, San Jose, CA, USA) and then protein was subjected to coomassie blue staining. The differentially-expressed bands were cutout and digested. Digests were analyzed by high performance liquid chromatography coupled with hybrid quadrupole-time of flight (LCMS-Q-TOF) at the Shenzhen Huada Genomics Institute using a TripleTOF 5600 System (AB SCIEX, Concord, ON) and data was processed and analyzed by Shenzhen Huada Genomics Institute (Shenzhen, China).

### RNA interference and plasmid transfection

siRNA oligos were synthesized by GenePharma (Shanghai, China). According to the manufacturer's instructions, RNA interference and plasmid transfection assays were performed using X-tremeGene siRNA transfection reagent and Lipofectamin^TM^ 3000 transfection reagent (Thermo Fisher Scientific, San Jose, CA, USA), respectively. The sequences of siRNA oligos were shown in Supplementary Table S2.

### Xenograft

Animal experiments were performed according to our previously published work 13 with slight modification, which was approved by the Animal Policy and Welfare Committee of Shantou University Medical College. Six-week-old male BALB/c nude (nu/nu) mice were provided by Beijing Weitonglihua Company in China and 2 mg/ml doxycycline and 10 mg/ml sucrose were added to drinking water before pLKO-Tet-On shRNA cell transplantation and maintained through all lifetime.Tet-On-mediated KYSE510 or KYSE150-shRNA1-circ-231, shRNA2-circ-231 and shRNA-Scramble cells were individually and subcutaneously injected into the right flanks of each mouse (5.0 × 10^6^ cells / flank) after the mice were anesthetized with an isoflurane/propylene glycol mixture. After one week, the tumor volume was measured every two days and tumors were collected and weighed at three weeks after all mice being euthanized by inhalation of CO_2_.

### Construction and pulldown of RNA secondary structure of 5′ UTR

To construct RNA secondary structure, DNA oligoes for the synthesis of 5′ UTR of TPI were synthesized and T7 RNA polymerase binding site sequence was added to 5′ end of the sense strand as follows: 5′-gatcactaatacgactcactatagggaga gcgcagacactgaccttcagcgcctcggctccagcgcc-3′ (Sense); 5′-GGCGCTGGAGCCGAGGCGCTGAAGGTCAGTGTCTGCGC-3′ (Antisense). The random sequence was used as negative control: 5′-gatcactaatacgactcactatagggaga GGCCACCGGTCCCCCACGGCATCCAGCGTCCGCAGTTA-3′ (Sense); 5′-TAACTGCGGACGCTGGATGCCGTGGGGGACCGGTGGCC-3′ (Antisense). The oligoes were annealed and then used as DNA template for RNA synthesis *in vitro* by using T7 RNA polymerase as the above conditions. The synthesized RNA was purified and further fold by RNA structure buffer 16 and RNA pulldown was performed as the above conditions.

### Bioinformatics analysis

circRNAs from seven cell lines, including A549, BJ, Hela S3, HepG2, K562, MCF7, HEK293, were downloaded from circBase database (http://www.circbase.org/) 17 and the overlapped circRNAs in all the seven cell lines were counted by using R package gplots from R-project database 18. The overlapped circRNAs were individually retrieved for the number of eIF4A3 binding sites in the junction sequence of the circRNAs in Circinteractome database (https://circinteractome.nia.nih.gov/) 7. Minimum free energy (MFE) structure and Centroid secondary structure for all the sequence, including the upstream of the EGFP initiation codon of pcDNA3.1-EGFP and the sequence containing 5′ UTRs of TPI1, PRDX6 and SS1 inserted into upstream site of EGFP initiation codon, were predicted as Mfold program (http://rna.tbi.univie.ac.at/) 19.

### Statistical analysis

Statistical analyses were performed using SPSS 19.0 for Windows (IBM, Chicago, IL, USA) and comparisons of the means of data between two groups were performed using two-sided Student's *t*-tests. Values of *P* < 0.05 were considered statistically significant.

## Results

### circ-231 interacts with eIF4A3

According to circBase database (http://www.circbase.org/) 17, we knew about numerous circRNAs expressed in multiple cell lines including A549 (14,970), BJ (12,705), Hela S3 (15,195), HepG2 (14,024), K562 (27,307), MCF7 (5,332) and HEK293 (239). Based on R package gplots from R-project database 18, we concluded that 51 circRNAs were overlapped in total seven cell lines (**Fig. 1A and Supplementary Table S3**). The overlapped 51 circRNAs were retrieved for the number of the sites of eIF4A3 binding to circRNA junctions (**Fig. 1B**) in CircInteractome database (https://circinteractome.nia.nih.gov/) 7. CircInteractome database provides the computational tool to predict the interaction between RNA binding proteins (RBPs) and circRNAs and the database enables the prediction of 109 RBPs containing eIF4A3, binding to circRNAs. Our data showed that 51 circRNAs were overlapped in seven cell lines, including A549, BJ, Hela S3, HepG2, K562, MCF7 and HEK293, which enlightened us to utilize the database to predict the potential interaction between these 51 circRNAs and eIF4A3. Finally, according to the prediction results provided by the database, we found that the three circRNAs, including circ-231, hsa_circ_0000069 (defined as circ-069) and hsa_circ_0000284 (defined as circ-284), possessed the potential binding sites of eIF4A3 (**Fig. 1C**), indicating that the three circRNAs potentially interacts with eIF4A3. Next, to explore whether eIF4A3 interacts with these circRNAs, HEK293T cells, a model cell line, and KYSE150 and KYSE510 were selected and the levels of circ-231, circ-069 and circ-284, was individually measured by using qRT-PCR and the results showed that the expression of circ-284 was highest among these circRNAs (**Fig. 1D**). Subsequently, RNA immunoprecipitation assays for eIF4A3 were performed in HEK293T, the potential circRNAs were determined and the results showed that circ-231 interacted with eIF4A3 (**Fig. 1E**). Since the levels of circ-231 were less than circ-284 in HEK293T and only the interaction of circ-231 with eIF4A3 was observed, indicating the interaction was specific. We also proved that circ-231 did not interact with two other members of the DEAD box protein family eIF4AI/II in HEK293T and KYSE150 cells (**Fig. 1F**) and the interaction of circ-231 with eIF4A3 was further demonstrated with circ-231 pulldown assays (**Fig. 1G**). The above data demonstrates circ-231 interacts with eIF4A3.

### circ-231 is co-localized with eIF4A3 in cytoplasm

**Figure 2:**
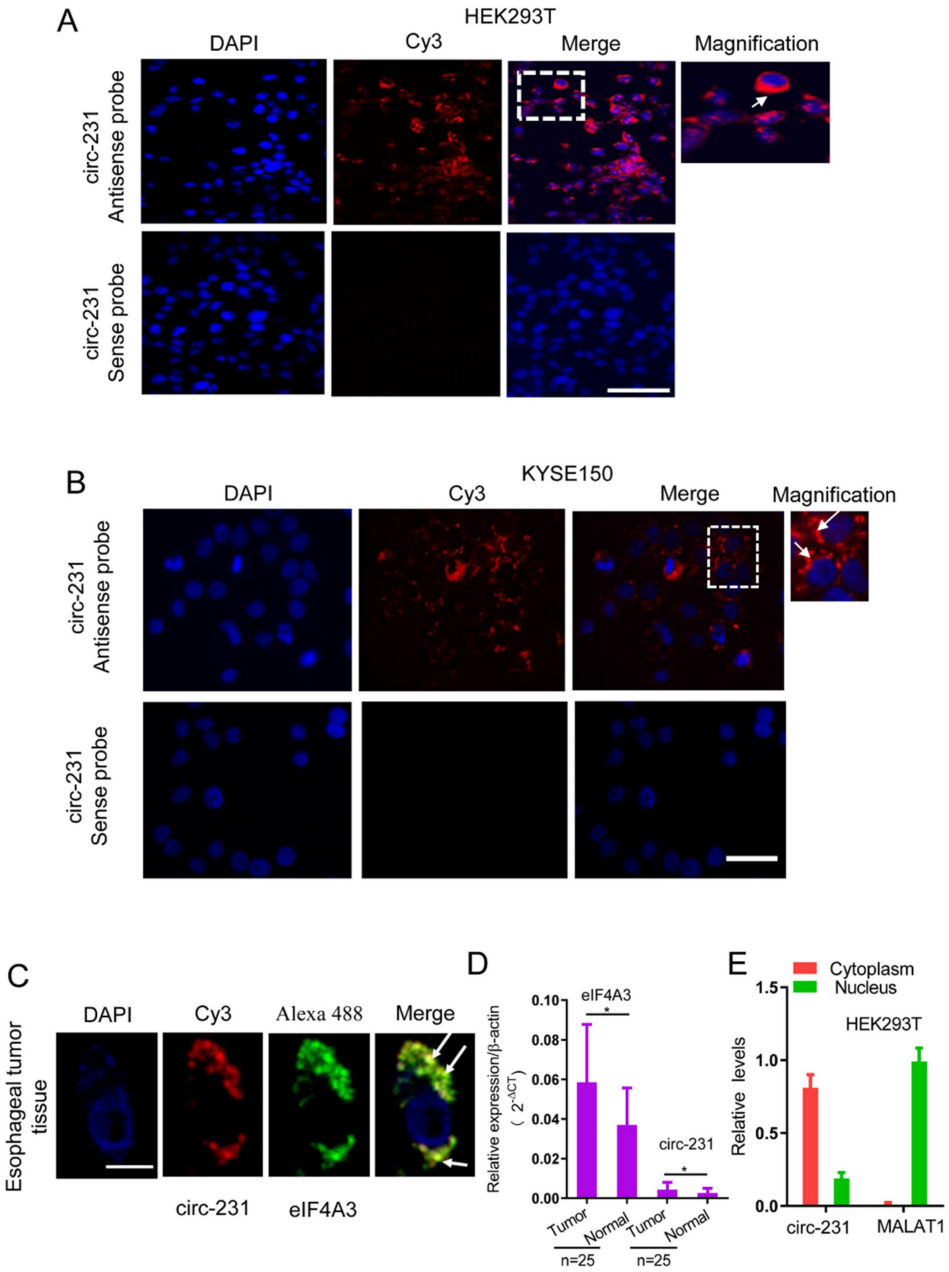
circ-231 was localized in cytoplasm and co-localized with eIF4A3 in HEK293T and ESCC cells. Fluorescence *in situ* hybridization assays for circ-231 was performed in HEK293T (**A**), KYSE150 cells (**B**). circ-231 antisense or sense probe (negative control) conjugated to biotin was detected using streptavidin-Cy3 (red). Cells were counterstained with DAPI (blue). Magnification, 40x. circ-231 is localized in cytoplasm in cells. circ-231 antisense or sense probe (negative control) conjugated to biotin was detected using streptavidin-Cy3 (red). Cells were counterstained with DAPI (blue). Magnification, 40 x. (**C**) Co-localization of circ-231 and eIF4A3 was performed in human esophageal tumor tissues. After biotin-coupled antisense probe for circ-231 was incubated with tumor tissue slides, mouse anti-eIF4A3 was simultaneously added to tissues at 37°C for 2 h and then Cy3-streptavidin and Alexa Fluor 488 dye-coupled goat anti-mouse IgG was added to tissues at room temperature for 1 h. Fluorescent signals were observed under 60 x magnification with 2 x optical magnification. (**D**) The relative levels of circ-231 and eIF4A3 transcripts in human esophageal tumor tissues (n=25) were detected using qRT-PCR. Values are mean ±SD. ^*^P˂0.05, ^**^P<0.01. β-actin used as internal control. (**E**) Cytoplasmic and nuclear RNAs were isolated from HEK293T cells and circ-231 was measured with qRT-PCR. A nuclear lncRNA MALAT1 transcript was used as control transcript. Values are mean ± SD.

To delineate the potential function of the interaction of circ-231 with eIF4A3, we detected the distribution of circ-231 in cells. The probe against the junction of circ-231 was designed and synthesized and the results of RNA FISH assays for circ-231 demonstrated that circ-231 was mainly localized in cytoplasm of HEK293T cells (**Fig. 2A**). This was further verified in KYSE150 and human esophageal tumor tissues (**Fig. 2B and Supplementary Fig. S1A**). In order to verify the specificity for the signal of the probe hybridization, circ-231 from shRNA1-circ-231 or shRNA-Scramble stable cell lines was determined with RNA FISH assays and the results established the specificity for the probe hybridization signal (**Supplementary Fig. S1B**). Moreover, fluorescent co-localization analysis demonstrated that circ-231 was co-localized with eIF4A3 in cytoplasm in human esophageal tumor cells (**Fig. 2C**). Importantly, upregulation of eIF4A3 and circ-231 in esophageal tumor tissues was observed, compared to adjacent normal tissues (**Fig. 2D**). Next, cytoplasm and nuclei were isolated and the results showed that cytoplasmic and nuclear extracts were successfully isolated because nuclear lncRNA MALAT1 transcript was predominantly localized in nucleus and in contrast, circ-231 was predominantly distributed in cytoplasm (**Fig. 2E**). Therefore, the above results suggest that circ-231 plays a role in cytoplasm in coordination with eIF4A3.

### circ-231 cooperates with eIF4A3 to promote protein expression, but not transcription

**Figure 3:**
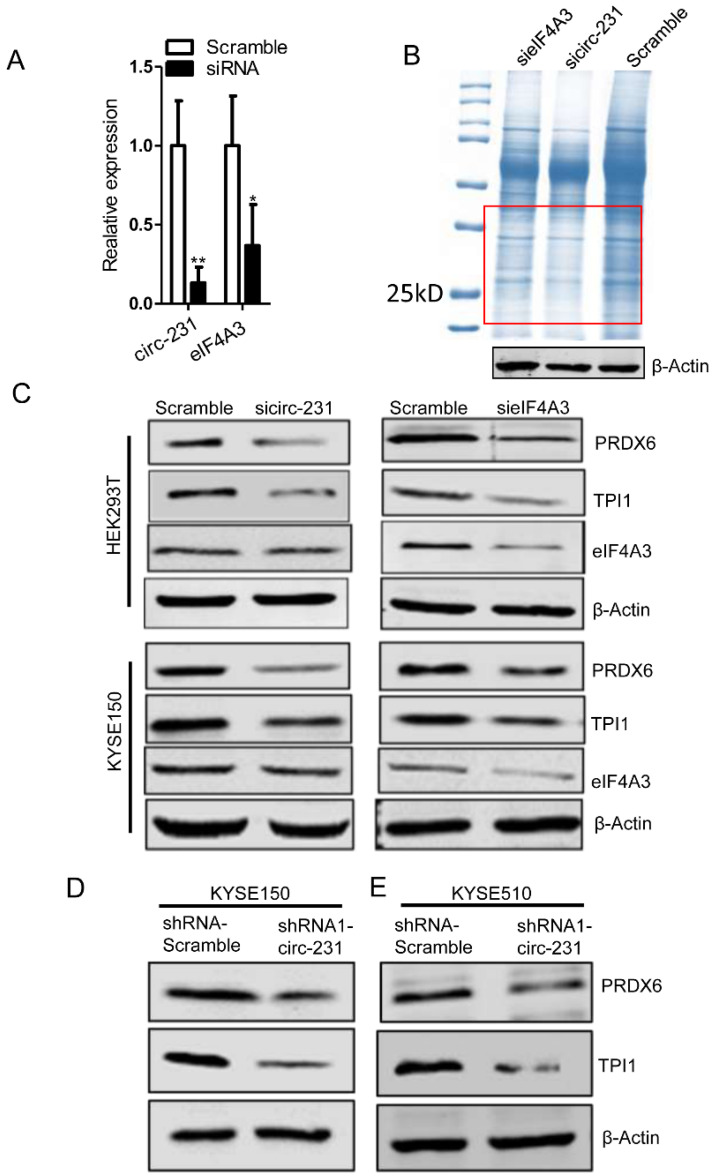
circ-231 cooperating with eIF4A3 promotes protein expression of PRDX6 and TPI1. (**A**) siRNAs against circ-231 or eIF4A3 were transfected into HEK293T and the relative levels of circ-231 and eIF4A3 transcripts were measured using qRT-PCR. Three independent experiments were performed. Values are mean ±SD. ^*^P<0.05, ^**^P<0.01. (**B**) After circ-231 or eIF4A3 knockdown, proteins were extracted and coomassie blue staining assays were performed and the differentially expressed protein bands were cutout and subjected to LCMS-Q-TOF analysis. Three independent experiments for coomassie blue staining were performed. (**C**) siRNAs against circ-231 or eIF4A3 were transfected into HEK293T or KYSE150 cells and protein levels of PRDX6, TPI1 and eIF4A3 were measured with western blot. In shRNA1-circ-231 or shRNA-Scramble stable cell lines, including KYSE150 (**D**) and KYSE510 (**E**) cells, protein levels of PRDX6, TPI1 and eIF4A3 were determined using western blot.

Since eIF4A3 is implicated in protein initial translation, we speculate that the interaction of circ-231 with eIF4A3 gives rise to the effect on protein level of target genes. To investigate the effect of the interaction of circ-231 with eIF4A3 on the protein expression of target genes, the coomassie brilliant blue staining assay was performed and three independent assays were performed after circ-231 or eIF4A3 knockdown in HEK293T cells (**Fig. 3A**) and the differentially-expressed protein bands shown as red box (**Fig. 3B**) were observed in each of experiments.

Therefore, the differentially-expressed protein bands simultaneously corresponding to both circ-231 and eIF4A3 knockdown were cutout and subjected to protein mass analysis (**Supplementary Table S4**). TPI1 and PRDX6 with higher scores were screened in order to further disclose the effect of the interaction of circ-231 with eIF4A3 on the expression of the two target genes. In HEK293T and KYSE150 cells, both circ-231 and eIF4A3 were down-regulated and the expression levels of TPI1 and PRDX6, including mRNA and protein, were measured. The results showed that mRNA levels of TPI1 and PRDX6 were not altered. However, protein levels of TPI1 and PRDX6 were decreased (**Fig. 3C**; **Supplementary Fig. S2A and B**). The expression of circ-231 parental gene ARHGAP12 was not altered after circ-231 knockdown in HEK293T cells, which excluded the effect of ARHGAP12 on the down-regulation of TPI1 and PRDX6 protein levels (**Supplementary Fig. S2C**). Also, circ-231 down-regulation caused the decrease of protein levels of TPI1 and PRDX6 in KYSE150 and KYSE510 expressing shRNA1-circ-231 in comparison with shRNA-Scramble. However, circ-231 knockdown did not alter the levels of TPI1 and PRDX6 mRNAs (**Fig. 3D and E; Supplementary Fig. S2D**). Moreover, circ-231 knockdown had no effect on the levels of eIF4A3 mRNA and protein (**Fig. 3C and D**; **Supplementary Fig. S2A, B and D**). In reverse, eIF4A3 knockdown did not result in alteration of circ-231 transcripts (**Supplementary. Fig S2A and B**). Therefore, circ-231 was implicated in protein expression of TPI1 and PRDX6 through the cooperation with eIF4A3.

### circ-231 in coordination with eIF4A3 promotes unwinding of secondary structure at 5′ end of mRNAs

**Figure 4:**
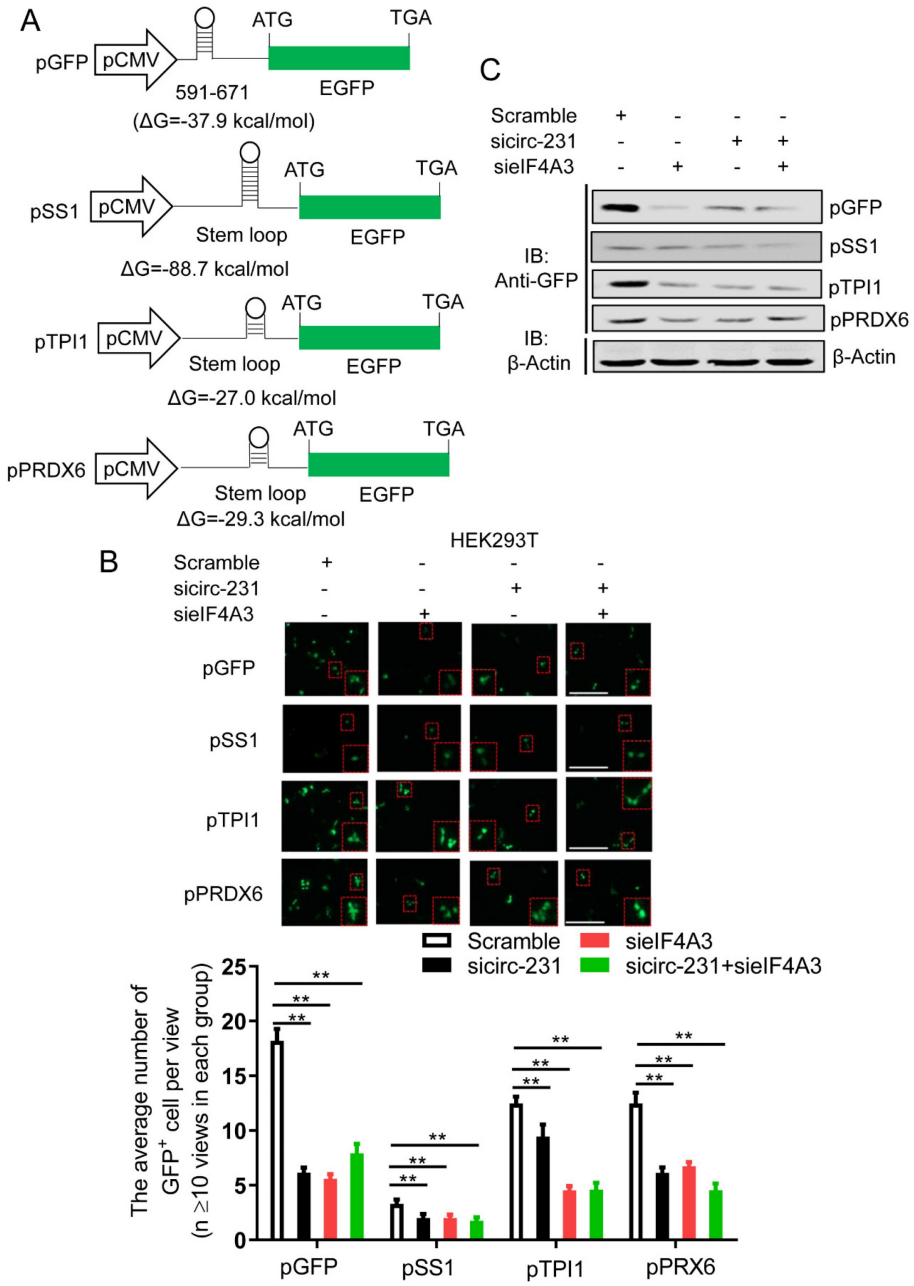
circ-231 in coordination with eIF4A3 promotes unwinding of secondary structure at 5′ end of mRNAs. (**A**) The schematic diagrams represent that those secondary structures with different free energy were individually inserted into Hind *III* sites upstream of initiation codon of pcDNA3.1-EGFP. (**B**) siRNAs against circ-231 or eIF4A3 were transfected or co-transfected into HEK293T cells and after 48 h, fluorescent signals of GFP were observed with fluorescent microscope. Magnification, 10x. The number of GFP^+^ cells from at least 20 views in each of groups were counted and analyzed. Values are mean ±SD. ^*^P<0.05, ^**^P<0.01. (**C**) siRNAs against circ-231 or eIF4A3 were transfected or co-transfected into HEK293T cells and after 48 h, the protein levels of GFP from every group were detected with western blot.

Considering that both circ-231 and eIF4A3 promote protein expression of TPI1 and PRDX6, but not transcription, we speculate that eIF4A3 might participate in unwinding of secondary structure at 5′ UTR of TPI1 and PRDX6 mRNAs through the interaction with circ-231. If thus, circ-231 or eIF4A3 knockdown results in the failure of unwinding of secondary structure, which deters the initial translation for mRNAs. However, how to indicate the change of secondary structure after circ-231 or eIF4A3 knockdown becomes important. In the study, we consider to use GFP as a tag to indicate the change of secondary structure of mRNA transcripts and we plan to insert secondary structure of 5′ UTR of TPI1 or PRDX6 mRNAs into upstream site from initial codon of GFP mRNAs and thereby translation of GFP-encoding mRNAs is regulated by the inserted secondary structure. Therefore, we hypothesize that the change of secondary structure will be manifested through measuring the change of GFP protein levels in cells. The levels of GFP protein will be decreased owning to secondary structure not being unwound after circ-231 or eIF4A3 knockdown and even in some cells, GFP fluorescence signal will be unavailable, which results in the decrease of the number of GFP^+^ cells. To verify our hypothesis, 5′ UTR of TPI1 and PRDX6 mRNAs and an artificial sequence containing stem-loop, as a positive control, were individually inserted into the Hind *III* site in upstream of initiation codon of pcDNA3.1-EGFP vector and then minimum free energy and secondary structure for all the inserted fragments in upstream of initiation codon of pcDNA3.1-EGFP were predicted by RNAfold server (http://rna.tbi.univie.ac.at/). We observed that there was a secondary structure in upstream of initiation codon of pcDNA3.1-EGFP vector with minimum free energy (ΔG = -37.9 kcal/mol) (**Fig. 4A and Supplementary Fig. S3**). In pSS1 group, secondary structure with lowest free energy (ΔG = -88.7 kcal/mol) was observed (**Fig. 4A and Supplementary Fig. S3**), indicating it is difficult for circ-231 and eIF4A3 to unwind the secondary structure. In fact, the results demonstrated our prediction. We found that circ-231 or eIF4A3 knockdown alone, as well as the combination of both circ-231 and eIF4A3 knockdown, resulted in a decrease of the number of GFP^+^ cells in each of groups, compared to the individual Scramble (**Fig. 4B**), which was further verified by western blot for determining GFP protein (**Fig. 4C**). The above results demonstrate that eIF4A3, depending on the interaction with circ-231, specifically promotes unwinding of secondary structure at 5′ UTR of TPI1 and PRDX6 mRNAs.

### STAU1 recruits eIF4A3 through interacting with circ-231 to mediate mRNA translation, but not transcription

**Figure 5:**
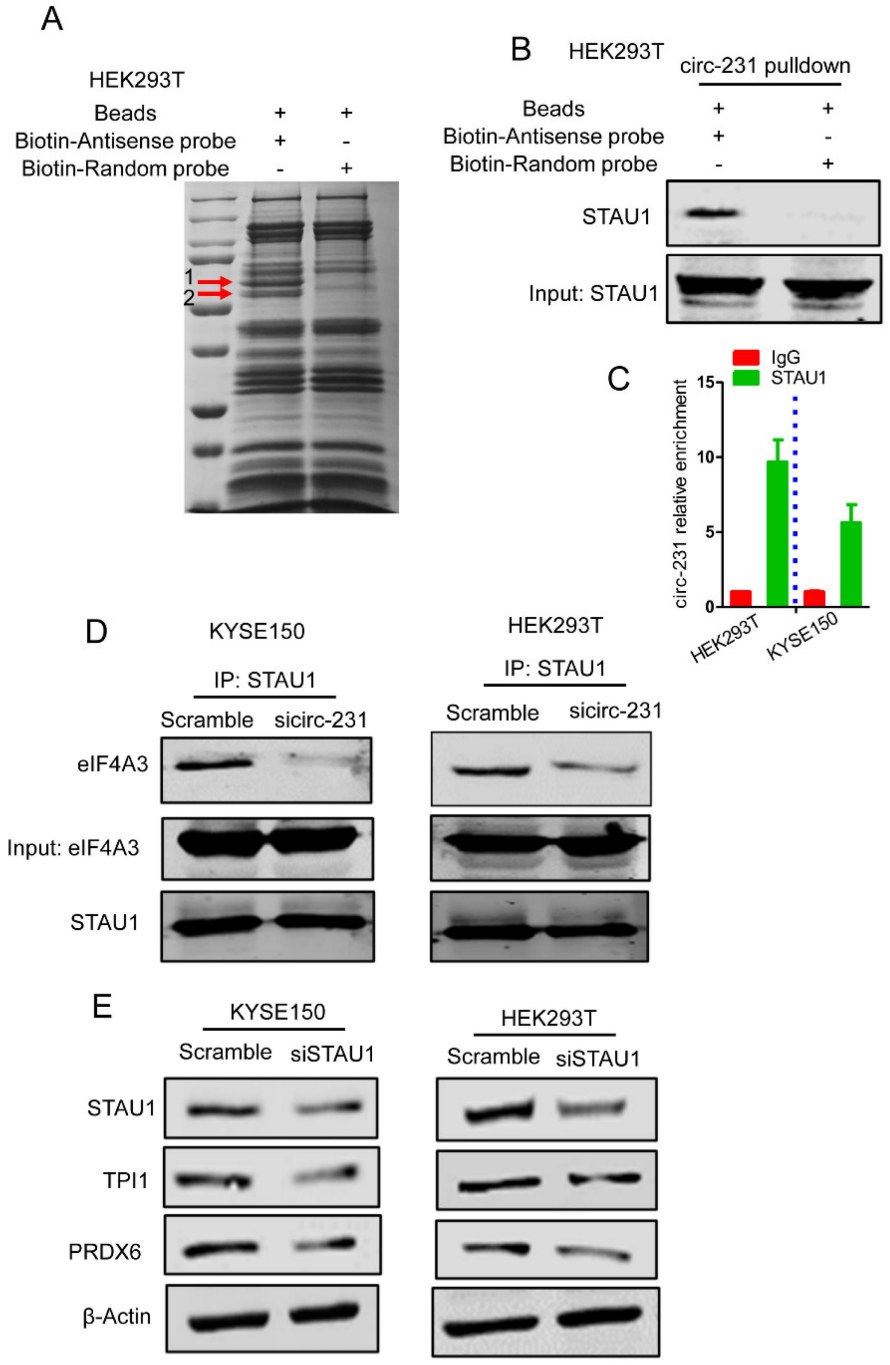
circ-231 promotes the interaction of eIF4A3 with STAU1. (**A**) circ-231 pulldown assays were performed. Biotin-labeled DNA probe complementary to junction sequence of circ-231 or random probe was incubated with HEK293T cell extracts at 4°C overnight and the biotin-labeled probes were pulled-down by streptavidin-coupled magnetic beads and the immunoprecipitated complex was subjected to SDS-PAGE electrophoresis and differential bands were dissected and identified by Mass analysis. (**B**) circ-231 pulldown was performed in HEK293T cells and STAU1 was determined with western blot. (**C**) RNA immunoprecipitation was performed using anti-STAU1 antibody in HEK293T and KYSE150 cells and RNA was extracted from the immunoprecipitated complex and circ-231 was determined using qRT-PCR. Values are mean ±SD. Two independent experiments were performed. (**D**) After circ-231 knockdown in HEK293T or KYSE150 cells, co-immunoprecipitation for STAU1 was performed and eIF4A3 was determined with western blot. (**E**) STAU1 was down-regulated in HEK293T or KYSE150 cells and the levels of TPI1 and PRDX6 protein were determined with western blot.

To further examine the mechanism of circ-231 interacting with eIF4A3 to unwind the secondary structure at 5′ end of mRNAs, circ-231 in HEK293T cells was pulled-down and compared to control, the different protein bands were dissected and subjected to Mass analysis (**Fig. 5A, band 1 and 2**), as indicated in **Supplementary Table S5**. We next ask which protein would be chosen during both circ-231 and eIF4A3 modulating protein synthesis. The protein would be chosen if it simultaneously meets the following criteria: 1) It is an RNA-binding protein. 2) Since eIF4A3 could bind to 5′UTR to regulate mRNA translation 6, it should be a protein involved in mRNA translation if the protein interacting with circ-231 and eIF4A3. 3) Proteins that participate in pre-mRNA alternative splicing should be excluded. 4) RNA helicases should be diminished because herein eIF4A3 is considered as a helicase. Based on the above four criteria, we screened each of proteins from the results of Mass analysis according to the published articles and found that STAU1 caught our attention. STAU1, as a double-strand RNA binding protein, binds to 5′UTR and participates in RNA translation 20. We hypothesize that STAU1 binds to 5′UTR and then eIF4A3 is recruited to bind to 5′UTR of TPI1 and PRDX6 mRNAs through circ-231 modulating the interaction of STAU1 with eIF4A3. Therefore, after circ-231 pulldown, STAU1 protein was proved to interact with circ-231 (**Fig. 5B**) and STAU1 RIP assays also proved that circ-231 interacted with STAU1 (**Fig. 5C**). Subsequently, circ-231 was down-regulated in KYSE150 or HEK293T cells and then the interaction of eIF4A3 with STAU1 was determined. The results showed that the interaction of eIF4A3 with STAU1 was decreased in response to circ-231 knockdown (**Fig. 5D**).

To interrogate the direct or indirect interaction for circ-231, eIF4A3 and STAU1, the Flag-labeled full-length and deleted mutant vectors of eIF4A3 were firstly individually transfected into HEK293T cells and then co-IP assays were performed using mouse anti-STAU1 antibody and Flag tags were detected with western blot to observe which domain of eIF4A3 interacted with STAU1. The results showed that both Flag-eIF4A3-wt and Flag-eIF4A3-1 interacted with STAU1, although the interaction of Flag-eIF4A3-1 with STAU1 was weaker than that of Flag-eIF4A3-wt, suggesting the ATP-binding domain of eIF4A3 interacted with STAU1 (**Supplementary Fig. S4A**). Next, to explore which domain of STAU1 interacts with eIF4A3, we conducted co-IP assays using rabbit anti-HA antibody after the HA-labeled full-length and deleted mutant vectors of STAU1 being individually transfected into HEK293T cells and eIF4A3 was measured using western blot. The results showed that, although there was weak interaction of eIF4A3 with HA-STAU1-2 (STAU1 C-end), the interaction of eIF4A3 with HA-STAU1-wt and HA-STAU1-1 (RNA binding domain) was obvious, indicating that eIF4A3 interacted with RNA-binding domain of STAU1 (**Supplementary Fig. S4B**). We also conducted RIP assays using anti-Flag or HA antibodies after Flag-or HA-labeled vectors with circ-231 expression vectors being individually transfected into HEK293T cells to observe which domain of eIF4A3 and STAU1 interacted with circ-231. The results of RIP assays showed that the obvious interaction between Flag-eIF4A3-wt and circ-231 was observed, compared to Flag-eIF4A3-1 and Flag-eIF4A3-2 (**Supplementary Fig. S4C**), suggesting that full-length of eIF4A3 might form a specific spatial structure indispensable for the interaction with circ-231. In contrast, there was obvious interaction of HA-STAU1-wt, HA-STAU1-1 with circ-231, indicating that RNA binding domain of STAU1 interacted with circ-231 (**Supplementary Fig. S4D**). Taken together, we speculate that circ-231, eIF4A3 and STAU1 forms the complex through direct interaction and STAU1 recruits eIF4A3 through interacting with circ-231.

Subsequently, we observed that the expressions of TPI1 and PRDX6 protein were also decreased after STAU1 was down-regulated in HEK293T or KYSE150 cell (**Fig. 5E**). However, the levels of TPI1 and PRDX6 mRNA were not altered (**Supplementary Fig. S5**). Therefore, circ-231 promotes the interaction of eIF4A3 with STAU1 to mediate the unwinding of 5′ UTR of TPI1 and PRDX6 mRNAs.

### STAU1 recruits eIF4A3 to unwind the secondary structure

**Figure 6:**
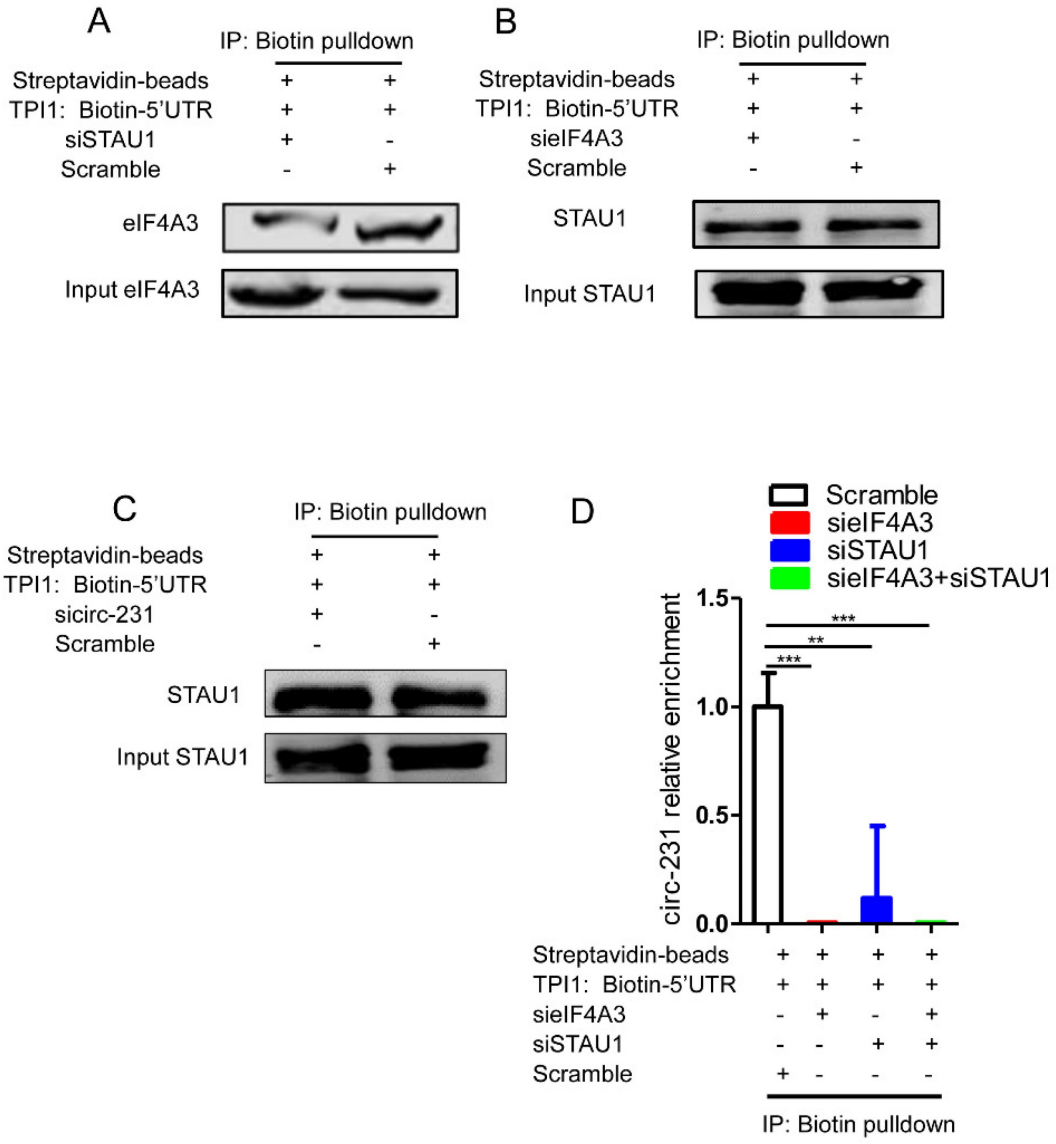
The binding of both eIF4A3 and circ-231 to secondary structure at 5′ UTR depends on STAU1. Biotinylated RNA from 5′ UTR of TPI1 mRNAs was synthesized *in vitro* transcription and allowed to form secondary structure and incubated with extracts from HEK293T cells with STAU1 (**A**), eIF4A3 (**B**) and circ-231 (**C**) knockdown, eIF4A3 or STAU1 from the immunoprecipitated complex was determined with western blot. (**D**) For circ-231, RNA from the immunoprecipitated complex was extracted and circ-231 was determined using qRT-PCR. Values are mean ±SD.

Since STAU1 is a double strand RNA-binding protein, we speculate that STAU1 binds to the secondary structure at 5′ end of mRNAs and then recruited eIF4A3 by circ-231 to unwind the secondary structure. Next, the secondary structure at 5′ end of TPI1 mRNAs were *in vitro* synthesized and labeled by biotin and incubated with protein extracts from HEK293T cells subjected to STAU1 knockdown. The results showed that eIF4A3 could bind to the synthesized secondary structure and the binding of eIF4A3 was decreased after STAU1 knockdown (**Fig. 6A**). However, circ-231 or eIF4A3 knockdown did not alter the binding of STAU1 to the *in vitro* synthesized secondary structure (**Fig. 6B and C**), validating that the binding of STAU1 did not depend on circ-231 and eIF4A3. We further found that eIF4A3 or STAU1 knockdown alone or the combination of the both resulted in the decrease of circ-231 binding to the secondary structure (**Fig. 6D**), suggesting that circ-231 did not directly bind to the secondary structure. Indeed, the binding of circ-231 to the secondary structure depended on both eIF4A3 and STAU1. Previous data showed that circ-231 promoted the interaction of eIF4A3 and STAU1 (**Fig. 5D**). Therefore, taken together, the data above demonstrates that circ-231 is used as a molecular scaffold to promote eIF4A3 interacting with STAU1 and thereby STAU1 recruits eIF4A3 to unwind the secondary structure at 5′ end of TPI1 and PRDX6 mRNAs.

### circ-231 promotes the migration and proliferation of ESCC and correlates with lymph node metastasis

**Figure 7:**
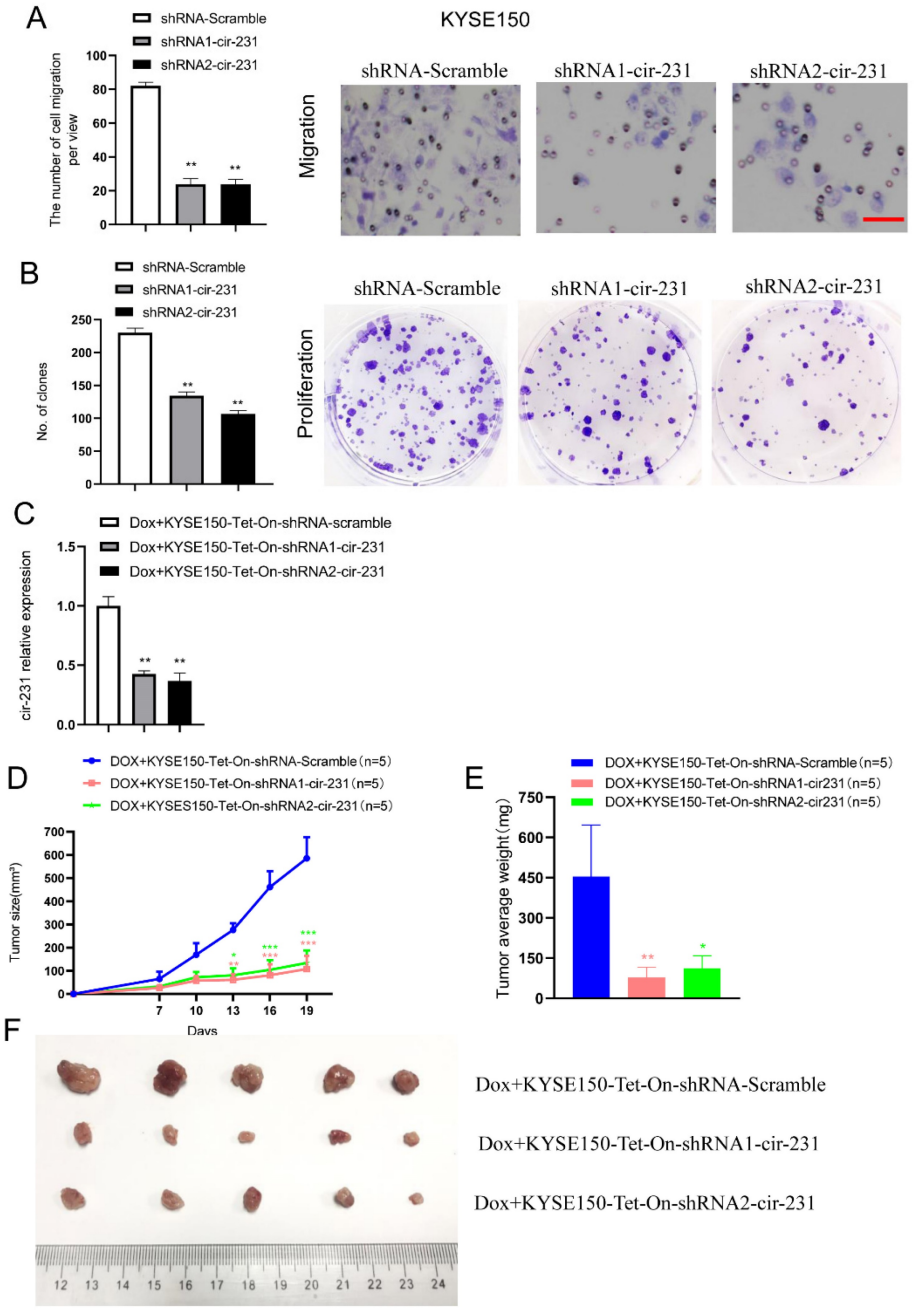
circ-231 promotes the migration and proliferation of human esophageal cancer cells. KYSE150 shRNA1-circ-231, shRNA2-circ-231 or shRNA-Scramble stable cell lines were subjected to starvation without serum culture medium for 18 h, the migration of cells was measured by transwell assays (**A**) and the proliferation of cells was detected by colony formation assays (**B**). Scale bar: 10 x. Values are mean ±SD. ^*^P<0.05, ^**^P<0.01. (**C-F**) KYSE150 Tet-on shRNA1-circ-231, shRNA2-circ-231 or shRNA-Scramble cell lines were individually subcutaneously injected into the right flank of each of six-week-old male BALB/c nude (nu/nu; n=5) mice (5.0 × 10^6^ cells / flank) and 2 mg/ml doxycycline and 10 mg/ml sucrose were added to drinking water before cell transplantation and maintained through all lifetime. The tumor volume was measured every two days and tumors were collected and weighed at three weeks after all mice being euthanized by the inhalation of CO_2._

To investigate the biological function of circ-231 in human esophageal cancer cells, KYSE150 shRNA-Scramble, shRNA1-circ-231 and shRNA2-circ-231 cell lines were established (**Supplementary Fig. S6**) and the migration capacities of the cells were measured. The results further validated that circ-231 knockdown led to the inhibition of the migration of cancer cells (**Fig. 7A**). Also, colony formation assays demonstrated that circ-231 knockdown inhibited the proliferation of cancer cells (**Fig. 7B**). Cancer xenograft mouse models for doxycycline-inducible Tet-On-shRNA1-circ-231, Tet-On-shRNA2-circ-231 and Tet-On-shRNA-Scramble in KYSE150 or KYSE510 cell lines further supported that tumor volume and average weight of Tet-On-shRNA1-circ-231 and shRNA2-circ-231 group were less than Tet-On-shRNA-Scramble (**Fig. 7C-F**; **Supplementary Fig. S7A-D**), suggesting that circ-231 knockdown inhibited the proliferation of cancer cells. The results above showed that circ-231 promoted the migration and proliferation of ESCC. We next sought to explore clinic implications of circ-231, since the up-regulated expression of circ-231 was observed in ESCC tissues, compared to paracancerous tissues. Due to lack of survival information for patients with ESCC, we failed to analyze the correlation of the expression levels circ-231 with overall survival and disease-free survival. However, it was intriguing that a large fraction of patients with highly-expressed circ-231 occurred to lymph node metastasis in comparison with patients without lymph node metastasis having lower expression of circ-231, insinuating that circ-231 may be an indicator for risk of lymph node metastasis for patients with ESCC. Additionally, there was a significant correlation for the expression levels of circ-231 with TNM, rather than age, gender, tumor size (**Supplementary Table S6**).

### circ-231 promotes migration and proliferation of ESCC through TPI1 and PRDX6

**Figure 8:**
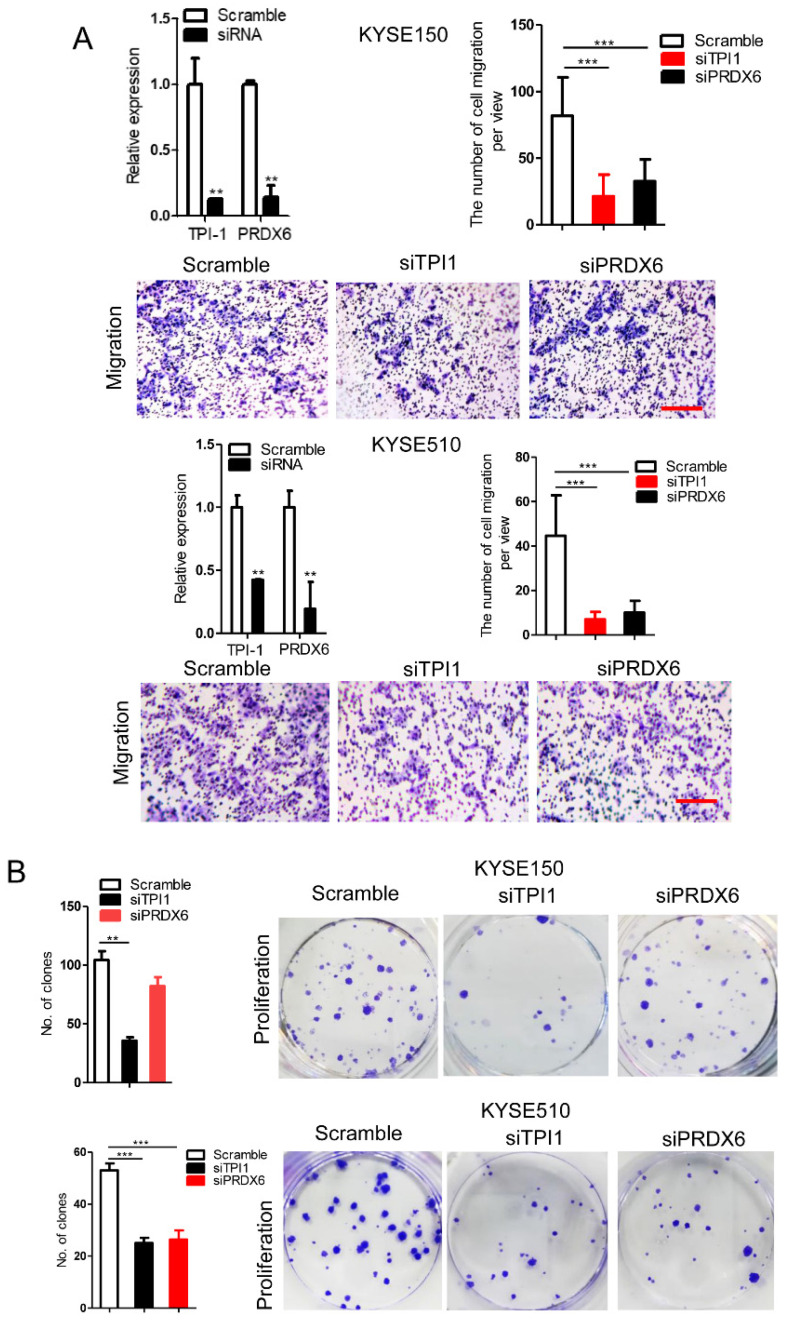
TPI1 and PRDX6 promote migration and proliferation of human esophageal cancer cells. After TPI1 or PRDX6 knockdown in KYSE510 and KYSE150 cells, the cells were subjected to starvation in serum-free culture medium for 18 h. Then, cell migration was measured by transwell assay (**A**), and cell proliferation was measured by colony formation assay (**B**). Scale bar: 10x. Values are mean ±SD. ^*^P<0.05, ^**^P<0.01,^ ***^P<0.001.

**Figure 9:**
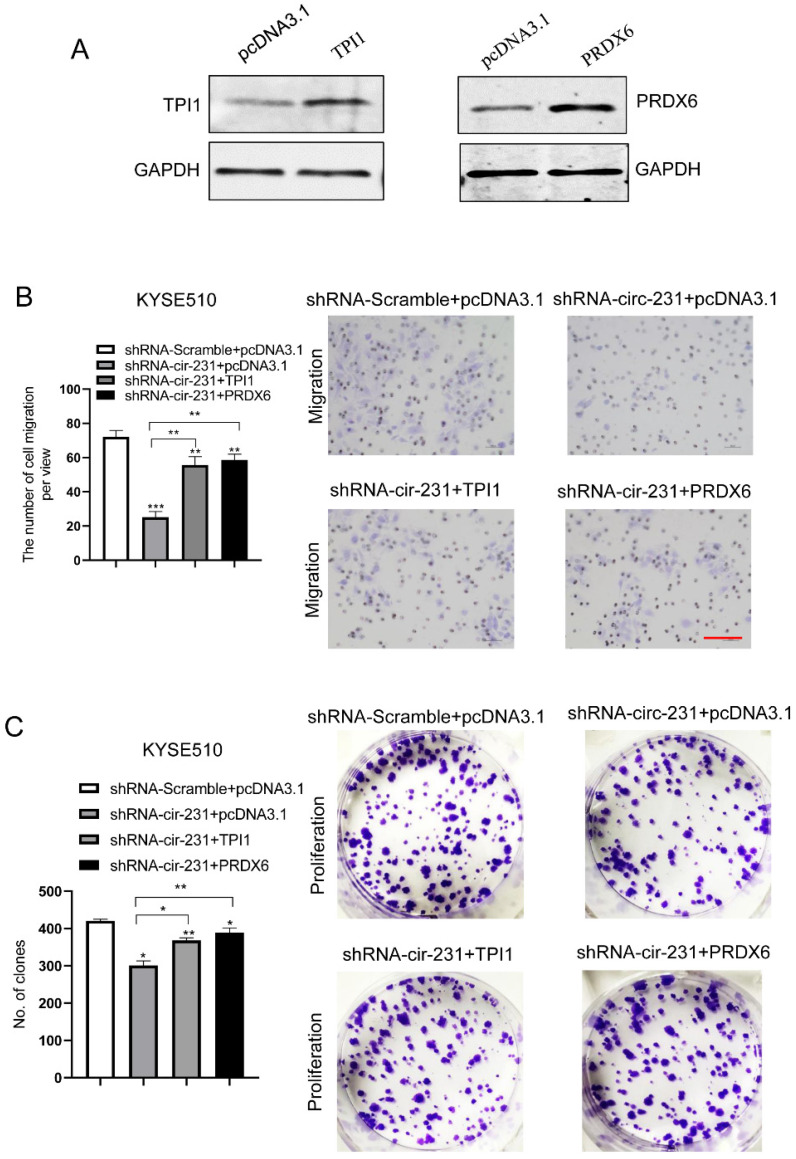
circ-231 promotes the migration and proliferation of human ESCC cells through TPI1 or PRDX6. (**A**) The expression vectors of pcDNA3.1, pcDNA3.1-TPI1 and pcDNA3.1-PRDX6 were individually transfected into KYSE510 cells and TPI1 and PRDX6 proteins were measured by using western blot. pcDNA3.1 was used as control. After the vectors of pcDNA3.1-TPI1 and pcDNA3.1-PRDX6 transfected into in KYSE510 shRNA-circ-231 cells, the cells were subjected to starvation in serum-free culture medium for 18 h. Then, cell migration was measured by transwell assay (**B**), and cell proliferation was measured by colony formation assay (**C**). pcDNA3.1 vectors were transfected into KYSE510 shRNA-Scramble and shRNA-circ-231 cells and used as control. Scale bar: 10x. Values are mean ±SD. ^*^P<0.05, ^**^P<0.01,^ ***^P<0.001.

We observed that TPI1 or PRDX6 knockdown resulted in the inhibition of the migration and proliferation of cancer cells and the knockdown inhibited the migration of cancer cells, although, in KYSE150, the proliferation of cancer cells was not altered after PRDX6 knockdown (**Fig. 8A and B**). Therefore, we speculate that circ-231 mediates migration and proliferation of ESCC through controlling TPI1 and PRDX6 protein synthesis. To verify the migration and proliferation of KYSE510 cells mediated by circ-231 through TPI1 and PRDX6 proteins, we performed the rescue assays by down-regulating circ-231 and simultaneously up-regulating TPI1 or PRDX6 expression (**Fig. 8A**) to observe the migration and proliferation capacities of KYSE510 cells. The results showed that the migration and proliferation capacities of KYSE510 cells were obviously rescued after circ-231 knockdown combined with TPI1 or PRDX6 up-regulation, compared to circ-231 knockdown group (**Fig. 9A-C**), although the migration and proliferation capacities of cells by rescue were still slightly lower than that of the control group. Therefore, we conclude that circ-231 is involved in migration and proliferation of cancer cells through mediating the synthesis of TPI1 and PRDX6 proteins.

## Discussion

**Figure 10:**
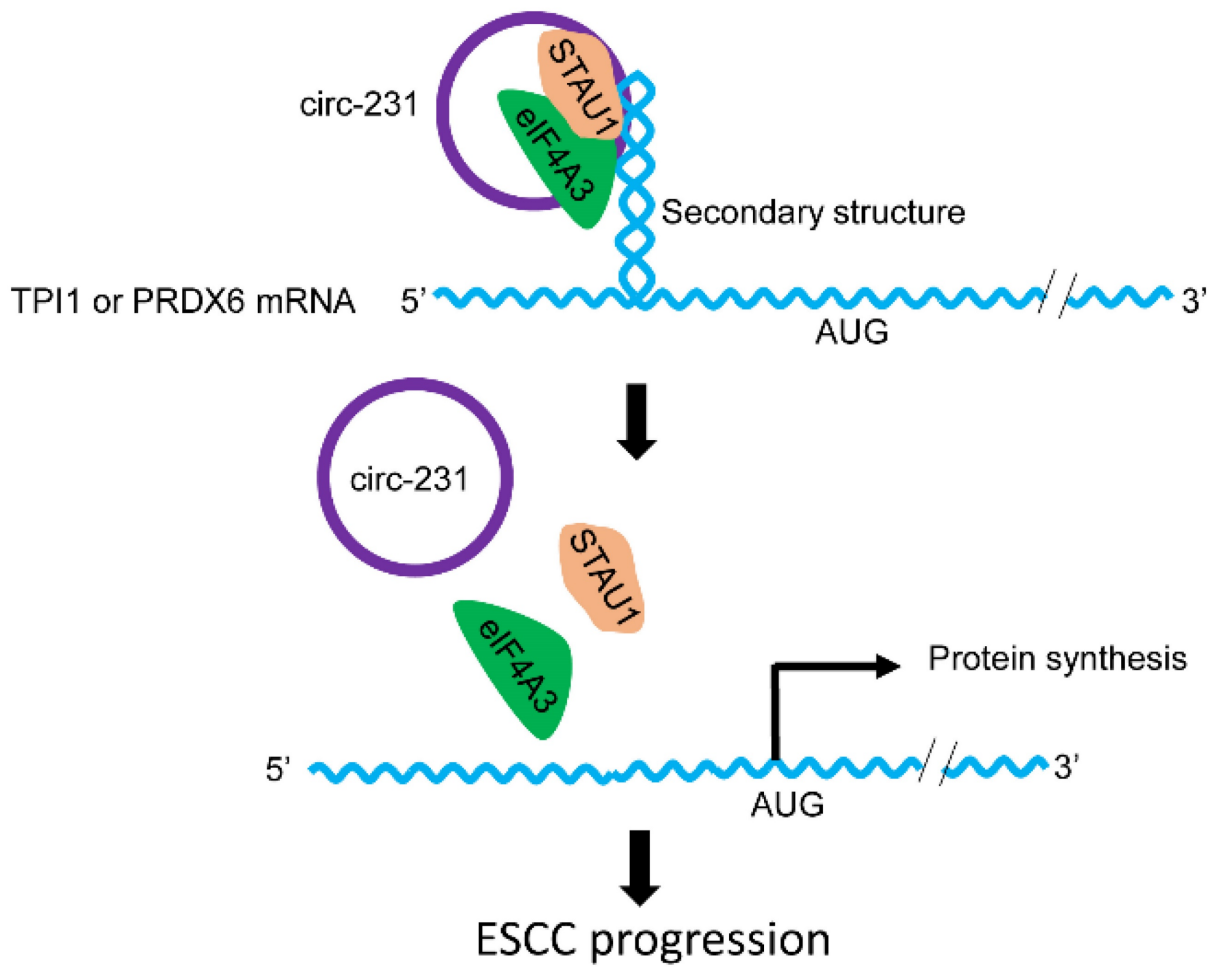
The schematic diagram for the interaction of circ-231 with eIF4A3 and STAU1 to promote unwinding of secondary structure in 5′ end of TPI1 and PRDX6 mRNAs, leading to ESCC progression.

Combined with previous reports 6, we propose a model for the mechanism by which eIF4A3 unwinds the secondary structure in ESCC. As indicated by **Fig. 10**, the newly synthesized mRNAs are exported into cytoplasm. At secondary structure of 5′ end, STAU1 is recruited and bound to double-strand RNA and subsequently, circ-231 promotes eIF4A3 to interact with STAU1 and thereby eIF4A3 unwinds the secondary structure. After the secondary structure is melted by the complex of circ-231 with eIF4A3 and STAU1, TPI1 and PRDX6 mRNA initiation translation is launched, promoting ESCC progression. Although the observed common mechanism was present in HEK293T cells and ESCC, circ-231 undertook a critical role in the mechanism and up-regulated expression of circ-231 in ESCC made it a vulnerable target for cancer therapy. Additionally, highly-expressed circ-231 in ESCC preferentially occurred to lymph node metastasis, indicative of feasibility to be identified as an indicator of risk of lymph node metastasis for patients with ESCC.

In the study, we observed that circ-231 promoted the interaction of eIF4A3 with STAU1 in HEK293T and ESCC cells. HEK293T was chosen in our study because we considered that it was a model cell line to be used for various functional studies according to the previously published articles. We further wondered whether circ-231 played the same role in other types of cancer cells and therefore, another cell line, hepatocellular carcinoma cell line HepG2 was used in our study to delineate the role of circ-231. The results for RNA immunoprecipitation assays showed that circ-231 interacted with eIF4A3 and STAU1 (**Supplementary Fig. S8A**). More importantly, co-IP assays showed that circ-231 knockdown led to the decrease of interaction between eIF4A3 and STAU1 (**Supplementary Fig. S8B and C**). Also, the results for the biological function of circ-231 showed that circ-231 knockdown caused the decrease of proliferation and migration capacities of HepG2 cells (**Supplementary Fig. S8D**). Therefore, we speculate that the above mechanism through which circ-231 mediates the interaction of eIF4A3 with STAU1 and biological function of circ-231 is common in multiple types of human cancer cells.

In the nucleus, nuclear cap-binding protein complex (CBC), which is composed of CBP80 and CBP20, binds to the 5′-cap structure of a newly synthesized mRNA. CBC-dependent translation initiation factor (CTIF) and eIF4A3, which are distributed in cytoplasm and nucleus, are subsequently recruited to 5′ end of mRNA to form CBC-CTIF-eIF4A3 complex in cytoplasm or nucleus 21, 22. However, in our present study, circ-231 and eIF4A3 were co-localized in cytoplasm (**Fig. 2C**). eIF4A3 was recruited by STAU1 through the interaction mediated by circ-231 to unwind secondary structure in 5′ UTR of mRNAs during the newly synthesized mRNA exported to cytoplasm. circ-231 knockdown resulted in eIF4A3 not melting secondary structure in 5′ UTR of mRNAs (**Fig. 5**), suggesting that RNA helicase activity of eIF4A3 depends on circ-231. In our present study, we report that circ-231, as a molecular scaffold, promotes the interaction between eIF4A3 and STAU1. And more importantly, STAU1 binds to double-stand RNA in 5′ UTR of mRNAs, and eIF4A3 is recruited to unwind the secondary structure through the interaction with STAU1 mediated by circ-231 (**Fig. 10**). Therefore, the knockdown of circ-231 eliminated the interaction of eIF4A3 with STAU1 and resulted in the inhibition of unwinding of secondary structure. However, it is still unclear whether the complex composed of circ-231, eIF4A3 and STAU1 first binds to the secondary structure or STAU1 first binds to the secondary structure followed by eIF4A3 mediated by circ-231.

Previous studies showed that TPI1 and PRDX6 protein played a critical role in cancer cells. For example, TPI1 catalyzed the isomerization of glyceraldehydes 3-phosphate (G3P) and dihydroxy-acetone phosphate (DHAP) in glycolysis and gluconeogenesis 23. PRDX6 participated in protection against oxidative injury and the suppression of PRDX6 increased the peroxide-induced cytotoxicity in breast cancer cells 20. TPT1 or PRDX6 protein up-regulation inhibited the apoptosis of cancer cells and promoted the resistance to chemotherapeutic drugs 25, 26. circ-231 promotes the unwinding of 5′ UTRs of TPI1 and PRDX6 mRNAs, which is associated with the migration and proliferation of cancer cells, suggesting that circ-231 plays a critical role in cancer cells. eIF4A3, as a nucleocytoplasmic shuttling protein, is located in the nucleus for pre-mRNA alternative splicing and in the cytoplasm for mRNA translation 1-4. eIF4A3 is up-regulated in several type of cancers and associated with tumor progression and oncogenesis 27. In the present study, both circ-231 and eIF4A3 were overexpressed in ESCC and participated in TPI1 and PRDX6 mRNA initial translation, suggesting that both circ-231 and eIF4A3 were involved in tumor progression via post-transcriptionally regulating the expression of TPI1 and PRDX6.

In summary, our results disclose a novel mechanism for eIF4A3 to unwind secondary structure in 5′ UTRs of mRNAs, which is significant for identifying new therapeutic target by interfering with mRNA initial translation in ESCC.

## Supplementary Material

Supplementary methods, figures and tables.

## References

[1] Wang Z, Murigneux V, Le Hir H (2014). Transcriptome-wide modulation of splicing by the exon junction complex. Genome Biol.

[2] Jiang Z, Tai Q, Xie X, Hou Z, Liu W, Yu Z (2021). EIF4A3-induced circ_0084615 contributes to the progression of colorectal cancer via miR-599/ONECUT2 pathway. J Exp Clin Cancer Res.

[3] Li Q, Lei C, Lu C, Wang J, Gao M, Gao W (2019). LINC01232 exerts oncogenic activities in pancreatic adenocarcinoma via regulation of TM9SF2. Cell Death Dis.

[4] Kanellis DC, Espinoza JA, Zisi A, Sakkas E, Bartkova J, Katsori AM (2021). The exon-junction complex helicase eIF4A3 controls cell fate via coordinated regulation of ribosome biogenesis and translational output. Sci Adv.

[5] Budiman ME, Bubenik JL, Driscoll DM (2011). Identification of a signature motif for the eIF4a3-SECIS interaction. Nucleic Acids Res.

[6] Choe J, Ryu I, Park OH, Park J, Cho H, Yoo JS (2014). eIF4AIII enhances translation of nuclear cap-binding complex-bound mRNAs by promoting disruption of secondary structures in 5'UTR. Proc Natl Acad Sci USA.

[7] Dudekula DB, Panda AC, Grammatikakis I, De S, Abdelmohsen K, Gorospe M CircInteractome (2016). A web tool for exploring circular RNAs and their interacting proteins and microRNAs. RNA Biol.

[8] Siegel RL, Miller KD, Jemal A (2020). Cancer statistics, 2020. CA Cancer J Clin.

[9] He AT, Liu J, Li F, Yang BB (2021). Targeting circular RNAs as a therapeutic approach: current strategies and challenges. Signal Transduct Target Ther.

[10] Zhang W, Wang B, Lin Y, Yang Y, Zhang Z, Wang Q, Zhang H, Jiang K, Ye Y, Wang S, Shen Z (2022). hsa_circ_0000231 Promotes colorectal cancer cell growth through upregulation of CCND2 by IGF2BP3/miR-375 dual pathway. Cancer Cell Int.

[11] Ji F, Lu Y, Chen S, Yu Y, Lin X, Zhu Y, Luo X (2021). IGF2BP2-modified circular RNA circARHGAP12 promotes cervical cancer progression by interacting m^6^A/FOXM1 manner. Cell Death Discov.

[12] Huang GW, Zhang YL, Liao LD, Li EM, Xu LY (2017). Natural antisense transcript TPM1-AS regulates the alternative splicing of tropomyosin I through an interaction with RNA-binding motif protein 4. Int J Biochem Cell Biol.

[13] Li CQ, Huang GW, Wu ZY, Xu YJ, Li XC, Xue YJ, et al Integrative analyses of transcriptome sequencing identify novel functional lncRNAs in esophageal squamous cell carcinoma Oncogenesis. 2017; 6: e297.

[14] Tripathi V, Ellis JD, Shen Z, Song DY, Pan Q, Watt AT (2010). The nuclear-retained noncoding RNA MALAT1 regulates alternative splicing by modulating SR splicing factor phosphorylation. Mol Cell.

[15] Huang R, Zhang Y, Han B, Bai Y, Zhou R, Gan G (2017). Circular RNA HIPK2 regulates astrocyte activation via cooperation of autophagy and ER stress by targeting MIR124-2HG. Autophagy.

[16] Wang P, Xue Y, Han Y, Lin L, Wu C, Xu S (2014). The STAT3-binding long noncoding RNA lnc-DC controls human dendritic cell differentiation. Science.

[17] Glažar P, Papavasileiou P, Rajewsky N (2014). circBase: a database for circular RNAs. RNA.

[18] Warnes G, Bolker B, Bonebakker L, Gentleman R, Huber W, Liaw A gplots: Various R programming tools for plotting data (comprehensive R archive network). 2016; R package version 3.0. 1.

[19] Gruber AR, Lorenz R, Bernhart SH, Neuböck R, Hofacker IL (2008). The vienna RNA websuite. Nucleic Acids Res.

[20] Dugré-Brisson S, Elvira G, Boulay K, Chatel-Chaix L, Mouland AJ, DesGroseillers L (2005). Interaction of Staufen1 with the 5' end of mRNA facilitates translation of these RNAs. Nucleic Acids Res.

[21] Kim KM, Cho H, Choi K, Kim J, Kim BW, Ko YG (2009). A new MIF4G domain-containing protein, CTIF, directs nuclear cap-binding protein CBP80/20-dependent translation. Genes Dev.

[22] Palacios IM, Gatfield D, St Johnston D, Izaurralde E (2004). An eIF4AIII-containing complex required for mRNA localization and nonsense-mediated mRNA decay. Nature.

[23] Shi Y, Vaden DL, Ju S, Ding D, Geiger JH, Greenberg ML (2005). Genetic perturbation of glycolysis results in inhibition of *de novo* inositol biosynthesis. J Biol Chem.

[24] Goncalves K, Sullivan K, Phelan S (2012). Differential expression and function of peroxiredoxin 1 and peroxiredoxin 6 in cancerous MCF-7 and noncancerous MCF-10A breast epithelial cells. Cancer Invest.

[25] Oesterreich S, Weng CN, Qiu M, Hilsenbeck SG, Osborne CK, Fuqua SA (1993). The small heat shock protein hsp27 is correlated with growth and drug resistance in human breast cancer cell lines. Cancer Res.

[26] Sinha P, Poland J, Kohl S, Schnölzer M, Helmbach H, Hütter G (2003). Study of the development of chemoresistance in melanoma cell lines using proteome analysis. Electrophoresis.

[27] Ye J, She X, Liu Z, He Z, Gao X, Lu L (2021). Eukaryotic Initiation Factor 4A-3: A Review of Its Physiological Role and Involvement in Oncogenesis. Front Oncol.

