# Supplementary material for: circular RNA circ-231 promotes protein biogenesis of TPI1 and PRDX6 through mediating the interaction of eIF4A3 with STAU1 to facilitate unwinding of secondary structure in 5′ UTR, enhancing progression of human esophageal squamous cell carcinoma (ESCC)
Source: J Cancer. 2024 Mar 11;15(9):2518–37. doi: 10.7150/jca.92578 (PMC10988296; doi:10.7150/jca.92578)
Supplement: Supplementary file 1 — Supplementary methods, figures and tables. [file jcav15p2518s1.pdf]

Supporting information

circular RNA circ-231 promotes protein biogenesis of TPI1 and PRDX6 through mediating the interaction of eIF4A3 with STAU1 to facilitate unwinding of secondary structure in 5′ UTR, enhancing progression of human esophageal squamous cell carcinoma (ESCC)

Guo-Wei Huang[1,2*], Ti-Qun Yang[1,2*], Qian-Qian Chen[1,2*], Xing-Mu Liu[3*], Ling-Hui Xie[1,2*], Wei Huang[1,2*], Xue-Ling Chen[1,2], Yi-Qun Geng[1,2], Jiang Gu [1,2&]

Supplementary Method DNA-sequencing for the PCR products of circ-231 and establishment for circ-231 expression vector.

Supplementary Table S1 Primers used in this study.

Supplementary Table S2 siRNAs used in this study.

Supplementary Table S3 Overlapping 51 circular RNAs in seven cell lines.

Supplementary Table S4 Mass spectroscopy analysis for circ-231 or eIF4A3 knockdown.

Supplementary Table S5 Mass spectrometry analysis for circ-231 pulldown.

Supplementary Table S6. Relationship between IGFBP5 expression and clinicopathological features in ESCC.

Supplementary Figure S1 circ-231 is localized in cytoplasm in human esophageal cancer cells.

Supplementary Figure S2 circ-231 knockdown does not alter the levels of transcripts of ARHGAP12, EIF4A3, TPI1, or PRDX6.

Supplementary Figure S3 Prediction of free energy (MFE) and partition function for secondary structure

Supplementary Figure S4 Identification of protein-protein or RNA-protein interaction domains for eIF4A3, STAU1 and circ-231.

Supplementary Figure S5 TPI1 and PRDX6 transcripts are not changed after STAU1 knockdown.

Supplementary Figure S6 The relative expression of circ-231 was measured by qRT-PCR.

Supplementary Figure S7. circ-231 promotes the migration and proliferation of human esophageal cancer cells.

Supplementary Figure S8 circ-231 promotes the interaction of eIF4A3 and STAU1 in HepG2 cell line and promotes migration and proliferation of HepG2 cells.

Supplementary Method

DNA-sequencing for the PCR products of circ-231 and establishment for circ-231 expression vector

The junction sequence of circ-231 (wt) was amplified and the PCR products were subsequently cloned into the pEASY-T vector (TransGen Biotech, Beijing, China) and sequenced by BGI (the Beijing Genomics Institute in Shenzhen, China). The Hind *III* recognition site sequence and the sequence homologous to the front circ-signal sequence of pHB-circBasic™ expression vector (Hanbio, Shanghai, China) were added to the 5′ end of the mature circ-231 (wt) and a rear circ-signal sequence with a Hind *III* recognition site sequence was added to the 3′ ends. The front circ-signal sequence was 5′-CGTACTAATGACTTTTTTTTTATACTTCAG-3′ and rear circ-signal sequence was 5′-GTAAGAAGCAAGGAAAAGAATTAGG-3′. The full-length sequence of linearized circ-231 containing a Hind *III* recognition site sequence and the front or rear circ-signal sequences was synthesized by Genewiz (Suzhou, China) and cloned into the pcDNA3.1 vector (Thermo Fisher Scientific, San Jose, CA, USA). The vector containing linearized circ-231 mature sequences was amplified by PCR with the forward primer for the front circRNA signal sequence and reverse primer for the rear circRNA signal sequence, and the PCR product was purified and then cloned into pHB-circBasic™ circRNA expression vector. The vector of pcDNA-circ-231-wt was transfected into cells and then transcribed and circularized by the complementarity between the front circ-signal and rear circ-signal sequences.

DNA-sequencing for circ-231 PCR products:

circ-231 mature Sequence

ATAGGACACGCTCATTTGGTCATTTTCCCGGTCCAGAGTTCTTGGATGTAGAGAAAAC
TAGCTTCTCCCAGGAACAATCTTGTGATTCCGCAGGAGAAGGCTCTGAAAGAATACA
TCAAGATTCTGAATCTGGTGATGAACTTAGCAGCAGCTCCACTGAACAGATAAGGGT
GATACTGGTGTAGATCTCCTTGTTCTAAGCTATATTCATCCAACAAGCCATAAAGCCA
TAATGTGGTATAACATCCTTTTTGAGAGGTGAATATTATTGAATGAAAATGGCTGACA
GAAGTGGGAAGATTATTCCAGGACAAGTGTATATTGAGGTGGAATATGATTATGAAT
ATGAAGCAAAGGACAGAAAGATTGTGATAAAACAAGGGGAGAGGTACATCTTGGTG
AAAAAGACCAATGATGACTGGTGGCAAGTCAAGCCAGATGAAAACTCCAAAGCGTTT
TATGTGCCAGCCCAGTATGTGAAGGAGGTCACGCGCAAAGCTCTCATGCCACCTGTT
AAGCAGGTAGCTGGTCTGCCAAATAACTCCACGAAAATAATGCAGAGTTTGCATCTT
CAGAGATCAACAGAAAATGTGAACAAATTGCCTGAGCTTTCAAGTTTCGGAAAGCCA
TCGTCATCTGTTCAAGGAACAGGTCTTATTCGTGATGCCAATCAGAATTTTGGACCCA
GTTATAATCAAGGTCAGACTGTCAACCTAAGCCTGGACCTGACCCATAATAACGGAA
AGTTTAACAATGACTCACATTCTCCTAAAGTTTCCAGCCAGA ⟶

(Blue and red fonts represent exon 3 and exon 2 of ARHGAP12, respectively. Black box represents the formed junction between exon 3 and exon 2 because of back splicing. Black arrow represents extension of exon 3 from the end with "CCAGCCAGA" to the underlined fonts).

Establishment of shRNA-circ-231 expression vectors

The sequences for shRNA-circ-231 or shRNA-Scramble cloned into AgeI and EcoRI sites of pLKO or pLKO-Tet-On empty vectors were as follows:

shRNA1-circ-231:

5'-CCGGGAACAGATAAGGGTTTAAACTCGAGTTTAAACCCTTATCTGTTCTTTTTGAATT-3'

shRNA2-circ-231:
5’-CCGGTGAACAGATAAGGGTTTAACTCGAGTTAAACCCTTATCTGTTCATTTTTGAAT
T-3’
shRNA-Scramble:
5’-
CCGGCCTAAGGTTAAGTCGCCCTCGCTCGAGCGAGGGCGACTTAACCTTAGGTTTTTG
AATT -3’

Table S1. Primers used in this study

Gene	Forward (5' to 3')	Reverse (5' to 3')
circ-0000069	GGACTTATGTAACCCACAGCCTTGC	CTTCATCTTCAACACCAGAGTCGTG
circ-0000231	ATAGGACACGCTCATTTGGTCATTT	TTCCCACTTCTGTCAGCCATTTTCA
circ-0000284	TCGGCAGCCTTACAGGGTTAAAGTA	ACACTACAAAAGGCACTTGACTGAG
TPI1	CAGACAAAGGTCATCGCAGATAACG	TCACAGAGCCTCCATAAATGATACG
PRX6	TCGTGTGGTGTTTGTTTTTGGTCCT	TTTGGCTTCTTCTTCAGGGATGGTT
ARHGAP12	GAGTAAGTGGCAACCTCGCAGTGAT	TTTGGCTTTGGCAACTGTCTGATTA
β-Actin	AGCGAGCATCCCCCAAAGTT	GGGCACGAAGGCTCATCATT
**Primers for circ-231 vector**		
circ-231	cgtactaatgacttttttttttatacttcagGTTTAAATGTGATACTGGTG	cctaattcttttccttgcttcttacCCTTATCTGTTCAGTGGAGC
**Primers for probe synthesis**		
circ-231 (Sense)	gatcactaatacgactcactatagggagaATAGGACACGCTCATTTGGTCATTT	TTCCCACTTCTGTCAGCCATTTTCA
circ-231 (Antisense)	ATAGGACACGCTCATTTGGTCATTT	gatcactaatacgactcactatagggagaTTCCCACTTCTGTCAGCCATTTTCA

Table S2. siRNAs used in this study

	Sense (5′-3′)	Antisense (5′-3′)
sicirc-231	UGAACAGAUAAGGGUUUAAdtdt	UUAAACCCUUAUCUGUUCAdtdt
sieIF4A3	AGUGGAAUUCGAGACCAGCdtdt	GCUGGUCUCGAAUUCCACUdtdt
siSTAU1	CCUGCAGUUGAACGAGUAAdtdt	UUACUCGUUCAACUGCAGGdtdt
siTPI1	GGGAGUCAGAUGAGCUGAUdtdt	AUCAGCUCAUCUGACUCCCdtdt
siPRDX6	UCCAGCAGAGAAGGAUGAAdtdt	UUCAUCCUUCUCUGCUGGAdtdt
Scramble	UUCUCCGAACGUGUCACGUdtdt	ACGUGACACGUUCGGAGAAdtdt

Table S3. Overlapping 51 circular RNAs from seven cell lines

hsa_circ_0000014	hsa_circ_0001361
hsa_circ_0000026	hsa_circ_0001380
hsa_circ_0000061	hsa_circ_0001414
hsa_circ_0000063	hsa_circ_0001436
hsa_circ_0000069	hsa_circ_0001461
hsa_circ_0000072	hsa_circ_0001535
hsa_circ_0000231	hsa_circ_0001558
hsa_circ_0000284	hsa_circ_0001615
hsa_circ_0000396	hsa_circ_0001661
hsa_circ_0000437	hsa_circ_0001663
hsa_circ_0000504	hsa_circ_0001666
hsa_circ_0000633	hsa_circ_0001727
hsa_circ_0000638	hsa_circ_0001730
hsa_circ_0000639	hsa_circ_0001746
hsa_circ_0000711	hsa_circ_0001756
hsa_circ_0000745	hsa_circ_0001801
hsa_circ_0000799	hsa_circ_0001821
hsa_circ_0000816	hsa_circ_0001897
hsa_circ_0000817	hsa_circ_0001900
hsa_circ_0000818	hsa_circ_0001904
hsa_circ_0000835	hsa_circ_0001922
hsa_circ_0000921	hsa_circ_0001136
hsa_circ_0001006	hsa_circ_0001147
hsa_circ_0001098	hsa_circ_0001251
hsa_circ_0001119	hsa_circ_0001278
	hsa_circ_0001360

Table S4. Mass spectrometry analysis for circ-231 or eIF4A3 knockdown

Accession	Score	Mass	Matches	Sequences	Description
P60174	3043	31057	155 (113)	15 (15)	Triosephosphate isomerase OS=Homo sapiens GN=TPI1 PE=1 SV=3
P30041	1032	25133	62 (47)	13 (12)	Peroxiredoxin-6 OS=Homo sapiens GN=PRDX6 PE=1 SV=3
P62241	1017	24475	62 (44)	11 (7)	40S ribosomal protein S8 OS=Homo sapiens GN=RPS8 PE=1 SV=2
P00492	717	24792	39 (27)	8 (7)	Hypoxanthine-guanine phosphoribosyltransferase OS=Homo sapiens GN=HPRT1 PE=1 SV=2
P50914	586	23531	24 (17)	5 (3)	60S ribosomal protein L14 OS=Homo sapiens GN=RPL14 PE=1 SV=4
P43487	492	23467	30 (22)	6 (6)	Ran-specific GTPase-activating protein OS=Homo sapiens GN=RANBP1 PE=1 SV=1
Q99714	431	27134	21 (17)	10 (10)	3-hydroxyacyl-CoA dehydrogenase type-2 OS=Homo sapiens GN=HSD17B10 PE=1 SV=3
Q9Y224	401	28165	16 (13)	7 (6)	UPF0568 protein C14orf166 OS=Homo sapiens GN=C14orf166 PE=1 SV=1
P14678	388	24765	32 (25)	5 (5)	Small nuclear ribonucleoprotein-associated proteins B and B' OS=Homo sapiens GN=SNRPB PE=1 SV=2
P21266	342	26998	12 (10)	6 (5)	Glutathione S-transferase Mu 3 OS=Homo sapiens GN=GSTM3 PE=1 SV=3
P60900	286	27838	17 (13)	8 (7)	Proteasome subunit alpha type-6 OS=Homo sapiens GN=PSMA6 PE=1 SV=1
P56537	279	27095	13 (12)	4 (4)	Eukaryotic translation initiation factor 6 OS=Homo sapiens GN=EIF6 PE=1 SV=1
P62906	197	24987	12 (6)	5 (3)	60S ribosomal protein L10a OS=Homo sapiens GN=RPL10A PE=1 SV=2
P22061	191	24792	16 (10)	7 (7)	Protein-L-isoaspartate(D-aspartate) O-methyltransferase OS=Homo sapiens GN=PCMT1 PE=1 SV=4
Q15631	166	26281	12 (6)	6 (3)	Translin OS=Homo sapiens GN=TSN PE=1 SV=1
P67870	165	25268	10 (6)	6 (4)	Casein kinase II subunit beta OS=Homo sapiens GN=CSNK2B PE=1 SV=1
P30084	153	31823	10 (7)	4 (4)	Enoyl-CoA hydratase, mitochondrial OS=Homo sapiens GN=ECHS1 PE=1 SV=4
P28161	128	25899	4 (4)	2 (2)	Glutathione S-transferase Mu 2 OS=Homo sapiens GN=GSTM2 PE=1 SV=2
Q8NBT2	124	22521	4 (3)	3 (3)	Kinetochore protein Spc24 OS=Homo sapiens GN=SPC24 PE=1 SV=2
Q9BV86	118	25770	9 (6)	5 (4)	N-terminal Xaa-Pro-Lys N-methyltransferase 1 OS=Homo sapiens GN=NTMT1 PE=1 SV=3
Q13162	115	30749	13 (7)	8 (6)	Peroxiredoxin-4 OS=Homo sapiens GN=PRDX4 PE=1 SV=1
O43809	106	26268	9 (6)	4 (3)	Cleavage and polyadenylation specificity factor subunit 5 OS=Homo sapiens GN=NUDT21 PE=1 SV=1
Q9NUJ1	101	34253	7 (5)	6 (5)	Mycophenolic acid acyl-glucuronide esterase, mitochondrial OS=Homo sapiens GN=ABHD10 PE=1 SV=1

Q92522	101	22474	2 (2)	1 (1)	Histone H1x OS=Homo sapiens GN=H1FX PE=1 SV=1
P19388	93	24650	4 (2)	2 (1)	DNA-directed RNA polymerases I, II, and III subunit RPABC1 OS=Homo sapiens GN=POLR2E PE=1 SV=4
O75489	91	30337	3 (2)	2 (2)	NADH dehydrogenase [ubiquinone] iron-sulfur protein 3, mitochondrial OS=Homo sapiens GN=NDUFS3 PE=1 SV=1
P18669	83	28900	8 (6)	2 (2)	Phosphoglycerate mutase 1 OS=Homo sapiens GN=PGAM1 PE=1 SV=2
Q8NCW5	83	31996	6 (5)	2 (2)	NAD(P)H-hydrate epimerase OS=Homo sapiens GN=APOA1BP PE=1 SV=2
P18124	81	29264	1 (1)	1 (1)	60S ribosomal protein L7 OS=Homo sapiens GN=RPL7 PE=1 SV=1
Q9NX63	80	26421	3 (3)	2 (2)	MICOS complex subunit MIC19 OS=Homo sapiens GN=CHCHD3 PE=1 SV=1
P08579	78	25470	3 (3)	1 (1)	U2 small nuclear ribonucleoprotein B'' OS=Homo sapiens GN=SNRPB2 PE=1 SV=1
P25787	76	25996	7 (2)	5 (1)	Proteasome subunit alpha type-2 OS=Homo sapiens GN=PSMA2 PE=1 SV=2
P30048	72	28017	2 (2)	1 (1)	Thioredoxin-dependent peroxide reductase, mitochondrial OS=Homo sapiens GN=PRDX3 PE=1 SV=3
P09211	67	23569	2 (2)	2 (2)	Glutathione S-transferase P OS=Homo sapiens GN=GSTP1 PE=1 SV=2
P51572	67	28031	2 (2)	1 (1)	B-cell receptor-associated protein 31 OS=Homo sapiens GN=BCAP31 PE=1 SV=3
P09936	64	25151	8 (2)	5 (2)	Ubiquitin carboxyl-terminal hydrolase isozyme L1 OS=Homo sapiens GN=UCHL1 PE=1 SV=2
P04183	61	26079	3 (1)	2 (1)	Thymidine kinase, cytosolic OS=Homo sapiens GN=TK1 PE=1 SV=2
O15173	56	23861	2 (1)	2 (1)	Membrane-associated progesterone receptor component 2 OS=Homo sapiens GN=PGRMC2 PE=1 SV=1
O75431	54	30086	2 (2)	2 (2)	Metaxin-2 OS=Homo sapiens GN=MTX2 PE=1 SV=1
Q5VTE0	54	50495	2 (2)	2 (2)	Putative elongation factor 1-alpha-like 3 OS=Homo sapiens GN=EEF1A1P5 PE=5 SV=1
P63104	48	27899	2 (2)	1 (1)	14-3-3 protein zeta/delta OS=Homo sapiens GN=YWHAZ PE=1 SV=1
P04406	44	36201	3 (1)	2 (1)	Glyceraldehyde-3-phosphate dehydrogenase OS=Homo sapiens GN=GAPDH PE=1 SV=3
Q6P587	43	25112	1 (1)	1 (1)	Acylpyruvase FAHD1, mitochondrial OS=Homo sapiens GN=FAHD1 PE=1 SV=2
Q99720	43	25169	2 (2)	2 (2)	Sigma non-opioid intracellular receptor 1 OS=Homo sapiens GN=SIGMAR1 PE=1 SV=1
Q9NVX0	42	27144	5 (2)	1 (1)	HAUS augmin-like complex subunit 2 OS=Homo sapiens GN=HAUS2 PE=1 SV=1
Q7Z4W1	40	26182	2 (1)	2 (1)	L-xylulose reductase OS=Homo sapiens GN=DCXR PE=1 SV=2
Q9BYD2	39	30395	1 (1)	1 (1)	39S ribosomal protein L9, mitochondrial OS=Homo sapiens GN=MRPL9 PE=1 SV=2
P78330	39	25163	6 (1)	3 (1)	Phosphoserine phosphatase OS=Homo sapiens GN=PSPH PE=1 SV=2
Q92979	38	26931	2 (1)	1 (1)	Ribosomal RNA small subunit methyltransferase NEP1 OS=Homo sapiens GN=EMG1 PE=1 SV=4

P19404	38	27659	2 (1)	1 (1)	NADH dehydrogenase [ubiquinone] flavoprotein 2, mitochondrial OS=Homo sapiens GN=NDUFV2 PE=1 SV=2
P30042	37	28495	3 (1)	2 (1)	ES1 protein homolog, mitochondrial OS=Homo sapiens GN=C21orf33 PE=1 SV=3
Q9Y2R9	37	28230	6 (1)	6 (1)	28S ribosomal protein S7, mitochondrial OS=Homo sapiens GN=MRPS7 PE=1 SV=2
Q9BTE7	35	27833	1 (1)	1 (1)	DCN1-like protein 5 OS=Homo sapiens GN=DCUN1D5 PE=1 SV=1
P05141	35	33059	1 (1)	1 (1)	ADP/ATP translocase 2 OS=Homo sapiens GN=SLC25A5 PE=1 SV=7
O14818	34	28041	2 (1)	2 (1)	Proteasome subunit alpha type-7 OS=Homo sapiens GN=PSMA7 PE=1 SV=1
Q9BUT1	34	27049	2 (1)	2 (1)	3-hydroxybutyrate dehydrogenase type 2 OS=Homo sapiens GN=BDH2 PE=1 SV=2
P84098	34	23565	1 (1)	1 (1)	60S ribosomal protein L19 OS=Homo sapiens GN=RPL19 PE=1 SV=1
Q9UFN0	33	28563	1 (1)	1 (1)	Protein NipSnap homolog 3A OS=Homo sapiens GN=NIPSNAP3A PE=1 SV=2
Q9NZ01	30	36410	5 (2)	1 (1)	Very-long-chain enoyl-CoA reductase OS=Homo sapiens GN=TECR PE=1 SV=1
Q13242	29	25640	1 (1)	1 (1)	Serine/arginine-rich splicing factor 9 OS=Homo sapiens GN=SRSF9 PE=1 SV=1
Q9Y3B3	29	25498	3 (2)	1 (1)	Transmembrane emp24 domain-containing protein 7 OS=Homo sapiens GN=TMED7 PE=1 SV=2
O43819	28	29962	1 (1)	1 (1)	Protein SCO2 homolog, mitochondrial OS=Homo sapiens GN=SCO2 PE=1 SV=3
Q13907	28	26645	6 (1)	3 (1)	Isopentenyl-diphosphate Delta-isomerase 1 OS=Homo sapiens GN=IDI1 PE=1 SV=2
Q9H6E4	25	26601	2 (1)	2 (1)	Coiled-coil domain-containing protein 134 OS=Homo sapiens GN=CCDC134 PE=1 SV=1
P82930	24	25692	4 (1)	2 (1)	28S ribosomal protein S34, mitochondrial OS=Homo sapiens GN=MRPS34 PE=1 SV=2
P62993	24	25304	4 (2)	3 (2)	Growth factor receptor-bound protein 2 OS=Homo sapiens GN=GRB2 PE=1 SV=1
Q9NY12	21	22505	1 (1)	1 (1)	H/ACA ribonucleoprotein complex subunit 1 OS=Homo sapiens GN=GAR1 PE=1 SV=1
P43235	19	37399	1 (1)	1 (1)	Cathepsin K OS=Homo sapiens GN=CTSK PE=1 SV=1

Table S5. Mass spectrometry analysis for circ-231 pulldown

Accession	Score	Mass	Matches	Sequences	Description
B4DUQ1	3227	48708	154(104)	15(13)	cDNA FLJ54552, highly similar to Heterogeneous nuclear ribonucleoprotein K OS=Homo sapiens PE=2 SV=1
V9HWE1	895	53676	64(33)	21(11)	Epididymis luminal protein 113 OS=Homo sapiens GN=HEL113 PE=2 SV=1
A0A024R3T8	688	113811	54(22)	17(7)	Poly [ADP-ribose] polymerase OS=Homo sapiens GN=PARP1 PE=4 SV=1
A0A0U1RRM4	362	62653	21(11)	7(6)	Polypyrimidine tract-binding protein 1 OS=Homo sapiens GN=PTBP1 PE=1 SV=1
A0A024RBE7	294	50696	19(10)	6(4)	Thymopoietin, isoform CRA_c OS=Homo sapiens GN=TMPO PE=4 SV=1
A0A0S2Z4Z9	282	54311	31(12)	9(6)	Non-POU domain containing octamer-binding isoform 1 (Fragment) OS=Homo sapiens GN=NONO PE=2 SV=1
B3KPS3	170	46725	8(5)	6(4)	Tubulin alpha chain OS=Homo sapiens PE=2 SV=1
B2R8Z8	101	69787	8(3)	6(3)	cDNA, FLJ94136, highly similar to Homo sapiens synaptotagmin binding, cytoplasmic RNA interacting protein (SYNCRIP), mRNA OS=Homo sapiens PE=2 SV=1
DKC1	96	58094	14(2)	7(2)	H/ACA ribonucleoprotein complex subunit 4 OS=Homo sapiens GN=DKC1 PE=1 SV=3
K2C6B	73	60315	5(2)	3(1)	Keratin, type II cytoskeletal 6B OS=Homo sapiens GN=KRT6B PE=1 SV=5
A8K4B4	65	49580	11(1)	6(1)	cDNA FLJ78441, highly similar to Homo sapiens nucleolar and spindle associated protein 1 (NUSAP1),mRNA OS=Homo sapiens PE=2 SV=1
P13645	62	59020	7(1)	6(1)	Keratin, type I cytoskeletal 10 OS=Homo sapiens GN=KRT10 PE=1 SV=6
Q9Y3I0	62	55688	9(1)	5(1)	tRNA-splicing ligase RtcB homolog OS=Homo sapiens GN=RTCB PE=1 SV=1
A0A024R4A0	59	76625	5(2)	4(2)	Nucleolin, isoform CRA_b OS=Homo sapiens GN=NCL PE=4 SV=1
I6L9E8	53	55595	5(1)	4(1)	Family with sequence similarity 98, member A OS=Homo sapiens GN=FAM98A PE=2 SV=1
M0QXS5	51	59292	3(1)	3(1)	Heterogeneous nuclear ribonucleoprotein L (Fragment) OS=Homo sapiens GN=HNRNPL PE=1 SV=1
A0A1W2PQ51	51	81091	2(1)	2(1)	Probable ATP-dependent RNA helicase DDX17 OS=Homo sapiens GN=DDX17 PE=1 SV=1
D3DTW3	50	48111	1(1)	1(1)	Insulin-like growth factor 2 mRNA binding protein 1 deltaN CRDBP OS=Homo sapiens GN=IGF2BP1 PE=2 SV=1
B4DEA3	49	42340	3(1)	3(1)	cDNA FLJ56531, highly similar to UV excision repair protein RAD23 homolog B OS=Homo sapiens PE=2 SV=1
B2R491	47	29807	4(1)	4(1)	40S ribosomal protein S4 OS=Homo sapiens GN=RPS4X PE=2 SV=1
A0A024R2K4	43	84372	3(1)	3(1)	Leucine rich repeat (In FLII) interacting protein 2, isoform CRA_b OS=Homo sapiens GN=LRRFIP2 PE=4 SV=1
A0A087X0X3	41	77746	7(0)	5(0)	Heterogeneous nuclear ribonucleoprotein M OS=Homo sapiens GN=HNRNPM PE=1 SV=1
K7EP90	41	48108	2(1)	2(1)	RNA-binding protein 42 OS=Homo sapiens GN=RBM42 PE=1 SV=1
A0A024RCZ1	40	57339	4(1)	4(1)	LEM domain containing 2, isoform CRA_a OS=Homo sapiens GN=LEMD2 PE=4 SV=1

Q9UMS4	38	55603	3(1)	2(1)	Pre-mRNA-processing factor 19 OS=Homo sapiens GN=PRPF19 PE=1 SV=1
A0A024RAC5	37	56790	2(1)	2(1)	Regulator of chromosome condensation 2, isoform CRA_a OS=Homo sapiens GN=RCC2 PE=4 SV=1
K7EKX4	32	5793	2(1)	2(1)	Polypyrimidine tract-binding protein 1 (Fragment) OS=Homo sapiens GN=PTBP1 PE=1 SV=1
M0R0M3	28	2892	6(0)	2(0)	Gamma-glutamylaminecyclotransferase (Fragment) OS=Homo sapiens GN=GGACT PE=1 SV=1
P13533	26	224394	2(0)	1(0)	Myosin-6 OS=Homo sapiens GN=MYH6 PE=1 SV=5
O95059	23	13912	6(0)	1(0)	Ribonuclease P protein subunit p14 OS=Homo sapiens GN=RPP14 PE=1 SV=3
K7ES89	23	14348	1(0)	1(0)	Dual-specificity protein phosphatase 3 (Fragment) OS=Homo sapiens GN=DUSP3 PE=1 SV=1
H3BPS0	22	8104	6(0)	1(0)	V-type proton ATPase subunit d 1 (Fragment) OS=Homo sapiens GN=ATP6V0D1 PE=4 SV=3
A0A024R1Z6	22	42122	3(0)	1(0)	Vesicle amine transport protein 1 homolog (T californica), isoform CRA_a OS=Homo sapiens GN=VAT1 PE=4 SV=1
H0Y3M2	21	21338	1(0)	1(0)	BUB3-interacting and GLEBS motif-containing protein ZNF207 (Fragment) OS=Homo sapiens GN=ZNF207 PE=1 SV=2
H0YGH7	20	47240	3(0)	1(0)	Phosphoinositide phospholipase C (Fragment) OS=Homo sapiens GN=PLCZ1 PE=1 SV=1
A0A0G2JN42	20	263143	2(0)	1(0)	Mucin-6 OS=Homo sapiens GN=MUC6 PE=1 SV=1
M0QX71	20	25632	3(0)	2(0)	Glutamate-rich WD repeat-containing protein 1 (Fragment) OS=Homo sapiens GN=GRWD1 PE=1 SV=1
Q6UXR4	19	35070	4(0)	1(0)	Putative serpin A13 OS=Homo sapiens GN=SERPINA13P PE=5 SV=1
A0A087X1A5	19	55140	1(0)	1(0)	Double-stranded RNA-binding protein Staufen homolog 1 OS=Homo sapiens GN=STAU1 PE=1 SV=1
M0QYA6	19	9654	1(0)	1(0)	Zinc finger protein 257 OS=Homo sapiens GN=ZNF257 PE=4 SV=1
A0A0C4DGF4	17	131662	1(0)	1(0)	Transient receptor potential cation channel subfamily M member 5 (Fragment) OS=Homo sapiens GN=TRPM5 PE=4 SV=1

Table S6. Relationship between circ-231 expression and clinicopathological features in ESCC

Characteristic		High expression of circ-231	Low expression of circ-231	*P*
n		16	9	
Age, n (%)	>60	8 (50%)	3 (33.33%)	0.4203
	<=60	8 (50%)	6 (66.67%)	
Gender, n (%)	Female	3 (18.75%)	4 (44.44%)	0.363
	male	11 (68.75%)	5 (55.56%)	
Tumor size, n (%)	>5 cm	15 (62.5%)	3 (33.33%)	0.2262
	<=5 cm	1 (37.5%)	6 (66.67%)	
Lymph node metastasis, n (%)	Yes	15 (93.75%)	2 (22.22%)	0.0005
	No	1 (6.25%)	7 (77.78%)	
TNM, n (%)				
T1-II	9	5 (31.25%)	8 (89.89%)	0.0112
TIII-IV	16	11 (68.75%)	1 (11.11%)	

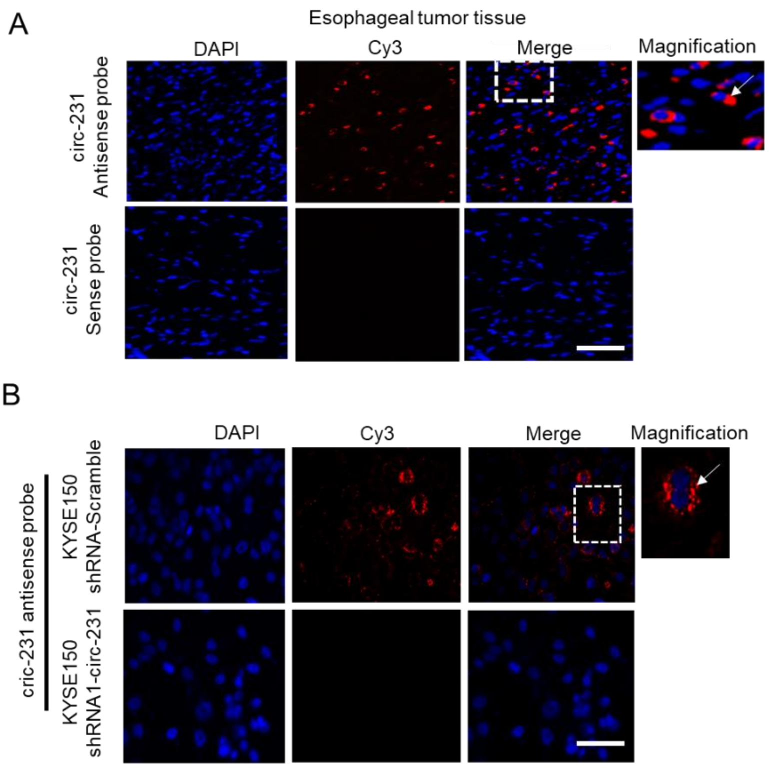

Figure S1 circ-231 is localized in cytoplasm in human esophageal cancer cells. Fluorescence in situ hybridization assays for circ-231 were performed in esophageal tumor tissue (A) and KYSE150 shRNA1-circ-231 or shRNA-Scramble stable cell lines (B). circ-231 antisense or sense probe (negative control) conjugated to biotin was detected using streptavidin-Cy3 (red). Cells were counterstained with DAPI (blue). Magnification, 40 x.

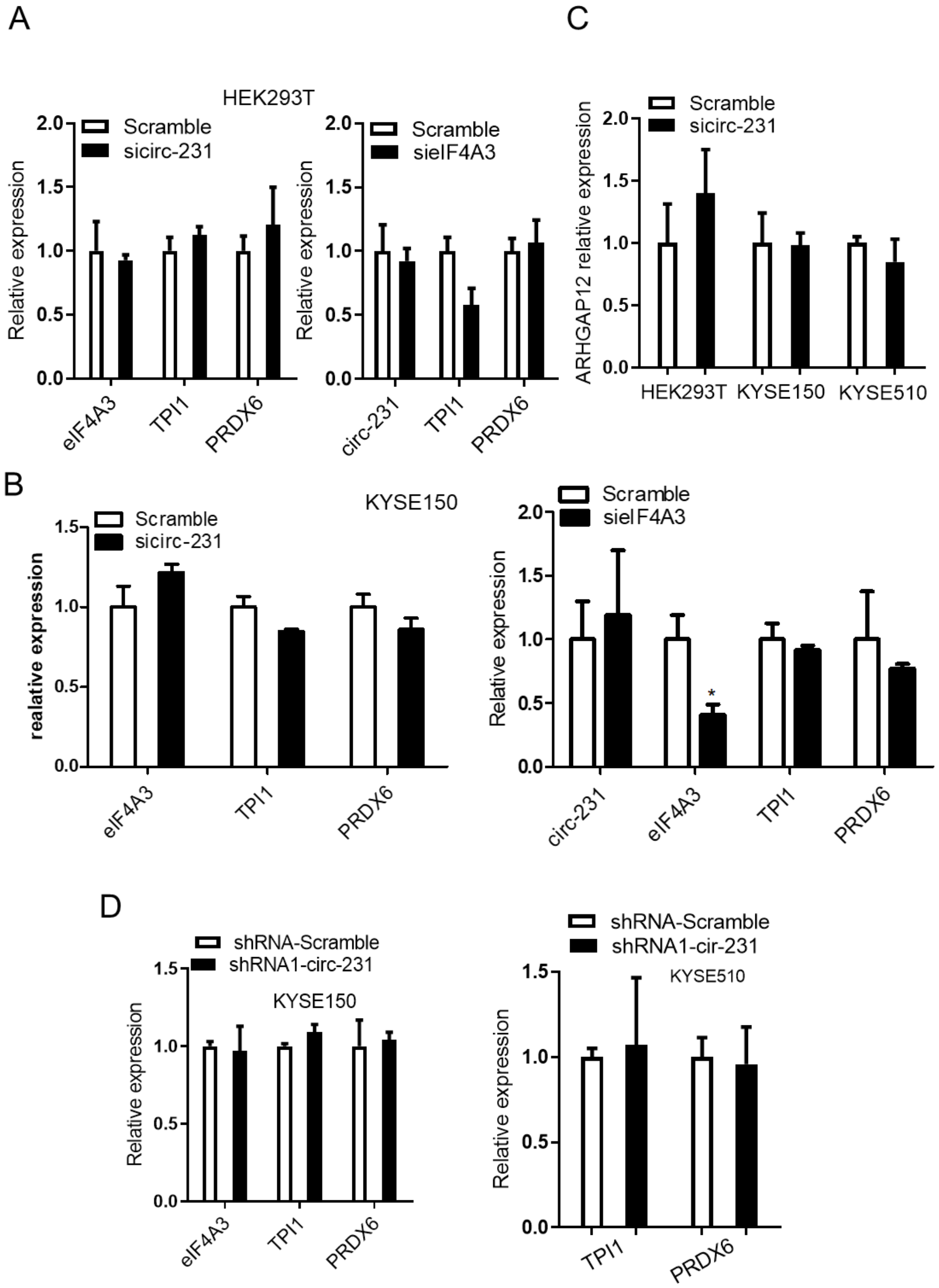

Figure S2. circ-231 knockdown does not alter the levels of transcripts for ARHGAP12, eIF4A3, TPI1, or PRDX6. After circ-231 was down-regulated in HEK293T (**A**) and KYSE150 cells (**B**), the relative expression of eIF4A3, TPI1 and PRDX6 was measured by qRT-PCR. **(C)** circ-231 parental gene ARHGAP12 was measured by qRT-PCR after circ-231 knockdown in HEK293T KYSE150 and KYSE510 cells. (**D**) After circ-231 was down-regulated in KYSE150 and KYSE510 shRNA-circ-231 or shRNA-Scramble stable cell lines, the relative expression of eIF4A3, TPI1 and PRDX6 was measured using qRT-PCR. Three independent experiments were repeated. Values are mean ±SD. $^{*}P<0.05$.

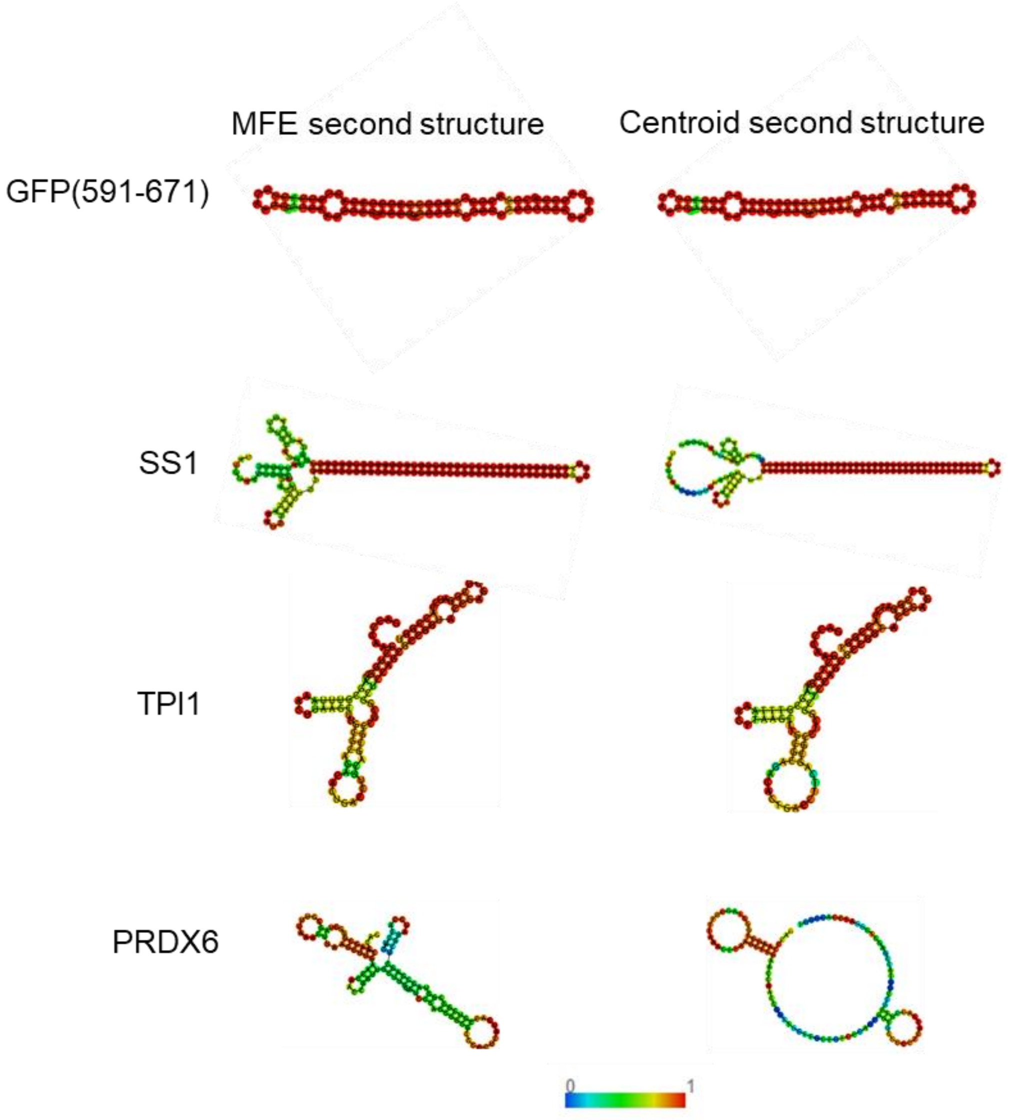

Figure S3. Prediction of free energy (MFE) and partition function for RNA secondary structure. Based on minimum free energy (MFE) and partition function, a stable stem-loop is predicted to have a stable double-stranded structure in the 5'UTRs of TPI1, PRDX6 mRNAs, as well as the synthesized fragment SS1 and the region from Nhe *I*/Age *I* upstream of the EGFP initiation codon in pcDNA3.1-EGFP. Color scale indicates the confidence for the prediction for each base with shades of red indicating strong confidence (http://rna.tbi.univie.ac.at/).

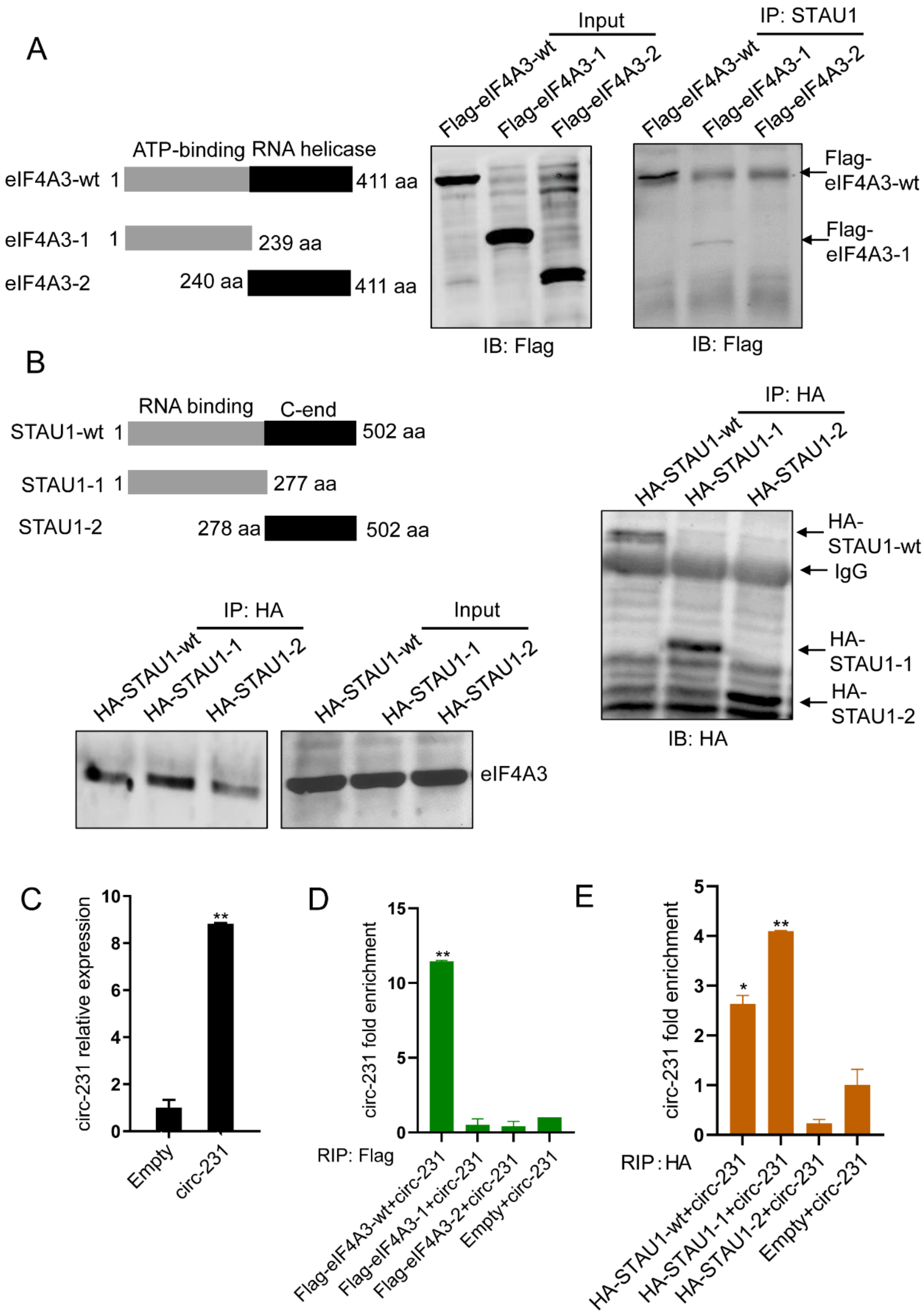

Figure S4. Identification of protein-protein or RNA-protein interaction domains for eIF4A3, STAU1 and circ-231. (**A**) Diagram shows full-length of eIF4A3 or deleted mutants. The vectors of Flag-eIF4A3-wt, Flag-eIF4A3-1(ATP-binding domain) and Flag-eIF4A3-2 (RNA helicase domain) were individually transfected into HEK293T cells and co-IP assays were performed using mouse STAU1 antibody and Flag tags

were measured using western blot. (**B**) Diagram shows full-length of STAU1 or deleted mutants. The vectors of HA-STAU1-wt, HA-STAU1-1 and HA-STAU1-2 were individually transfected into HEK293T cells and co-IP assays were performed using HA-tag antibody and both eIF4A3 and HA tags were detected using western blot. (**C**) The vector of circ-231 was constructed. Values are mean ±SD. $^{*}P<0.05$, $^{**}P<0.01$. The vector of circ-231 was co-transfected into HEK293T cells with the individual vectors of Flag-eIF4A3-wt, Flag-eIF4A3-1 and Flag-eIF4A3-2 (**D**) and the individual vectors of HA-STAU1-wt, HA-STAU1-1, HA-STAU1-2 (**E**) and Flag- or HA-RIP assays were performed and the relative enrichment of circ-231 was measured using qRT-PCR. Empty vector was used as control. Values are mean ±SD. $^{*}P<0.05$, $^{**}P<0.01$.

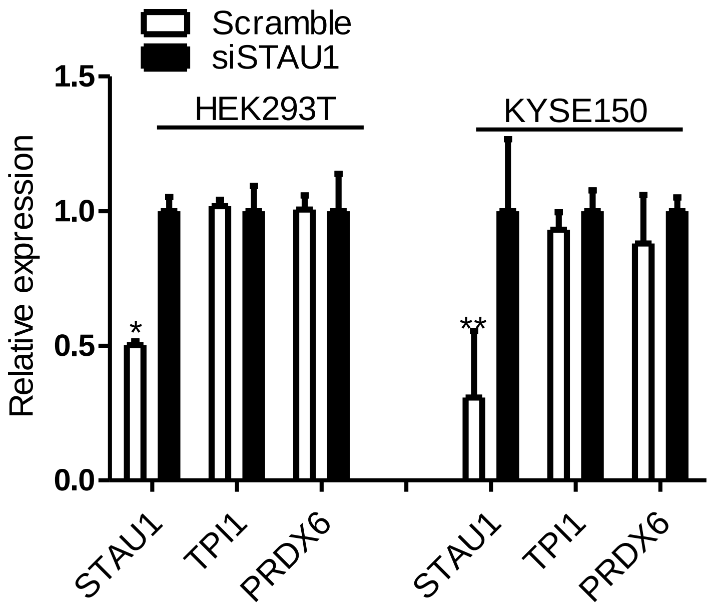

Figure S5. TPI1 and PRDX6 transcripts are not changed after STAU1 knockdown. **(A)** In HEK293T and KYSE150 cells, the levels of TPI1 and PRDX6 mRNA were determined using qRT-PCR after STAU1 knockdown. Values are mean ±SD. $^{*}P<0.05$, $^{**}P<0.01$.

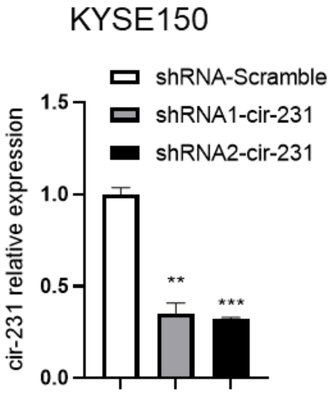

Figure S6. The relative expression of circ-231 was measured by qRT-PCR. KYSE150 shRNA1-circ-231, shRNA2-circ-231 and shRNA-Scramble stable cell lines were established and circ-231 relative expression was measured by using qRT-PCR. $^{*}P<0.05$, $^{**}P<0.01$, $^{***}P<0.001$.

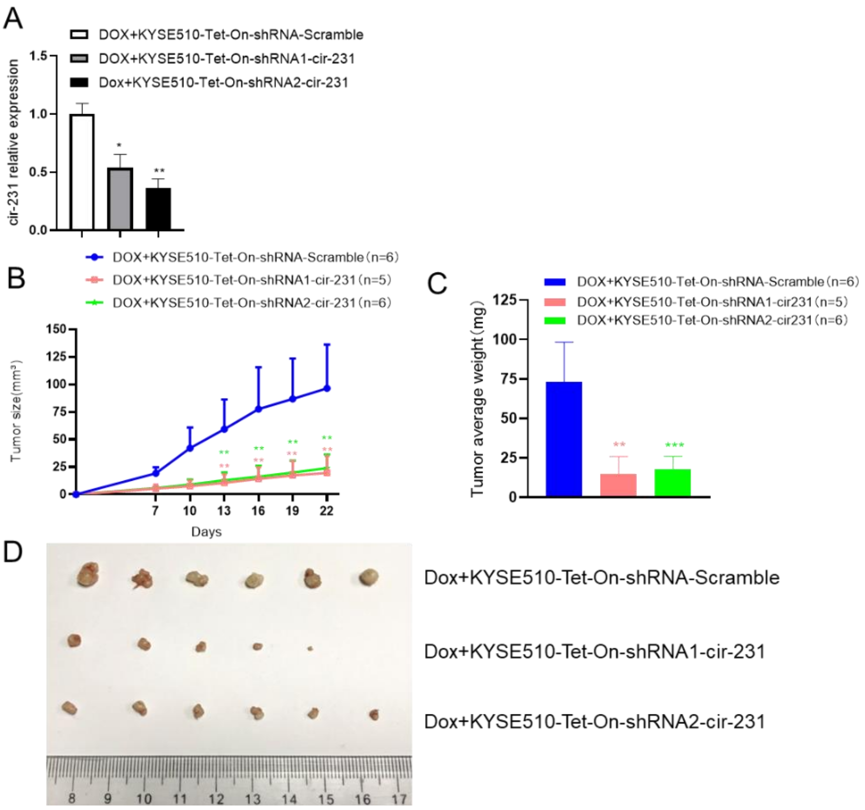

Figure S7. circ-231 promotes the migration and proliferation of human esophageal cancer cells. KYSE510 Tet-on shRNA1-circ-231, shRNA-circ-231 and shRNA-Scramble cell lines were established **(A)** and then individually subcutaneously injected into the right flank of each of six-week-old male BALB/c nude (nu/nu; n=6) mice (5.0 $\times 10^6$ cells / flank) and 2 mg/ml doxycycline and 10 mg/ml sucrose were added to drinking water before cell transplantation and maintained through all lifetime. The tumor volume was measured every two days **(B)** and tumors were collected and weighed **(C and D)** at three weeks after all mice being euthanized by the inhalation of CO_2. Values are mean ±SD. $^{*}P$<0.05, $^{**}P$<0.01, $^{***}P$<0.001.

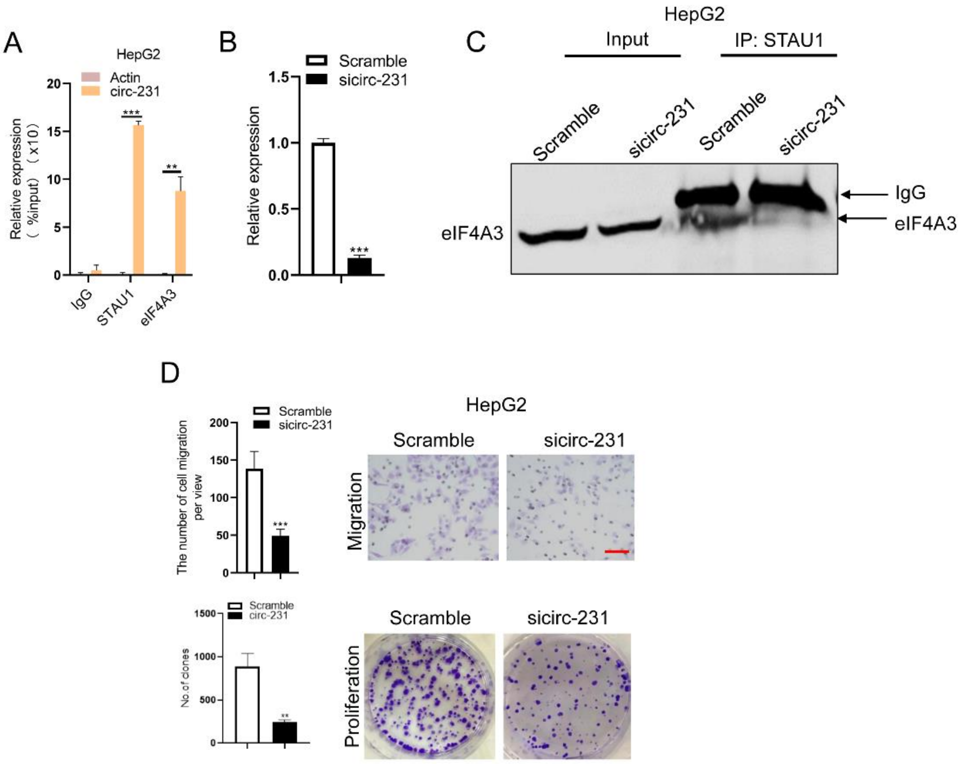

Figure S8. circ-231 promotes the interaction of eIF4A3 and STAU1 in HepG2 cell line and promotes migration and proliferation of HepG2 cells. (**A**) RIP assays were individually performed by using mouse anti-STAU1, mouse anti-eIF4A3 antibodies and mouse normal IgG and RNA was extracted from the immunoprecipitated complex and circ-231 was determined by using qRT-PCR. Actin was used as control. Values are mean ±SD. $^{*}P<0.05$, $^{**}P<0.01$, $^{***}P<0.001$. (**B and C**) circ-231 was determined by using qRT-PCR after siRNA against circ-231 or Scramble siRNAs transfected into HepG2 cells and co-IP to assess the interaction between eIF4A3 and STAU1 was performed by using mouse anti-STAU1 antibody and eIF4A3 was measured by western blot. Values are mean ±SD. $^{*}P<0.05$, $^{**}P<0.01$, $^{***}P<0.001$. (**D**) After circ-231 knockdown in HepG2 cells, the cells were subjected to starvation in serum-free culture medium for 18 h. Then, cell migration was measured by transwell assay and cell proliferation was measured by colony formation assay. Scale bar: 10x. Values are mean ±SD. $^{*}P<0.05$, $^{**}P<0.01$, $^{***}P<0.001$.
